# Enhancing severe hypoglycemia prediction in type 2 diabetes mellitus through multi-view co-training machine learning model for imbalanced dataset

**DOI:** 10.1038/s41598-024-69844-z

**Published:** 2024-09-30

**Authors:** Melih Agraz, Yixiang Deng, George Em Karniadakis, Christos Socrates Mantzoros

**Affiliations:** 1 Division of Applied Mathematics, Brown University, Providence, RI 02912 USA; 2 Department of Statistics, Giresun University, Giresun, 28200 Turkey; 3 School of Engineering, Brown University, Providence, RI 02912 USA; 4 Department of Computer and Information Science, College of Engineering, University of Delaware, Newark, DE 19716 USA; 5 Ragon Institute of Mass General, MIT and Harvard, Cambridge, MA 02142 USA; 6 Department of Endocrinology, Beth Israel Deaconess Medical Center, Harvard Medical School, Boston, MA 02215 USA

**Keywords:** Predictive medicine, Machine learning, Statistical methods, Data mining

## Abstract

Patients with type 2 diabetes mellitus (T2DM) who have severe hypoglycemia (SH) poses a considerable risk of long-term death, especially among the elderly, demanding urgent medical attention. Accurate prediction of SH remains challenging due to its multifaced nature, contributed from factors such as medications, lifestyle choices, and metabolic measurements. In this study, we propose a systematic approach to improve the robustness and accuracy of SH predictions using machine learning models, guided by clinical feature selection. Our focus is on developing long-term SH prediction models using both semi-supervised learning and supervised learning algorithms. Using the action to control cardiovascular risk in diabetes trial, which includes electronic health records for over 10,000 individuals, we focus on studying adults with T2DM. Our results indicate that the application of a multi-view co-training method, incorporating the random forest algorithm, improves the specificity of SH prediction, while the same setup with Naive Bayes replacing random forest demonstrates better sensitivity. Our framework also provides interpretability of machine learning models by identifying key predictors for hypoglycemia, including fasting plasma glucose, hemoglobin A1c, general diabetes education, and NPH or L insulins. The integration of data routinely available in electronic health records significantly enhances our model’s capability to predict SH events, showcasing its potential to transform clinical practice by facilitating early interventions and optimizing patient management. By enhancing prediction accuracy and identifying crucial predictive features, our study contributes to advancing the understanding and management of hypoglycemia in this population.

## Introduction

Type 2 diabetes mellitus (T2DM) results from either reduced insulin production, insulin resistance, or both. T2DM outnumbers both gestational diabetes and type 1 diabetes mellitus (T1DM) in prevalence, accounting for nearly 90% of all diagnosed cases^1^. Hypoglycemia can potentially have a critical impact on morbidity and mortality risk in T2DM^2^. From a clinical perspective, hypoglycemia can be divided into two categories based on standard criteria: mild hypoglycemia (MH) or severe hypoglycemia (SH). MH is typically defined by a blood sugar level of 70 mg/dl, while SH is defined by a blood sugar level of 54 mg/dl or lower and is determined based on whether the patient experiences loss of consciousness or needs medical assistance^3^. One critical area of risk prediction is the estimation of SH in diabetes, as it is marked by the need for immediate medical assistance and it is believed to be a strong risk factor of long-term mortality^4,5^. SH can lead to seizures, coma, and brain damage^6–9^, and sometimes it can be fatal. According to a recent analysis conducted by the Action to Control Cardiovascular Risk in Diabetes (ACCORD) study, the presence of SH with medical assistance was linked to a 50% higher likelihood of developing heart failure^10^, and youngs with T1DM may die from hypoglycemia in up to 10% of cases^11^. Therefore, predicting SH risks in advance is important to prevent future heart attacks and take precautions against the resulting impact. For this reason, the clinical motivation of the study is to help healthcare professionals evaluate SH risks, predict the SH events and take precautions, thus protecting patients from the side effects of SH events in the future.

Electronic health records (EHRs), which include comprehensive patient records such as demographics, laboratory results, diagnoses, and medical histories, are digitally maintained throughout the treatment or follow-up process^12,13^. As large datasets become increasingly available and computing resources become more powerful, complex analyses that were previously not possible with statistical methods have been done using machine learning (ML) techniques, enabling more accurate and effective predictions, particularly in the medical field.

**Figure 1:**
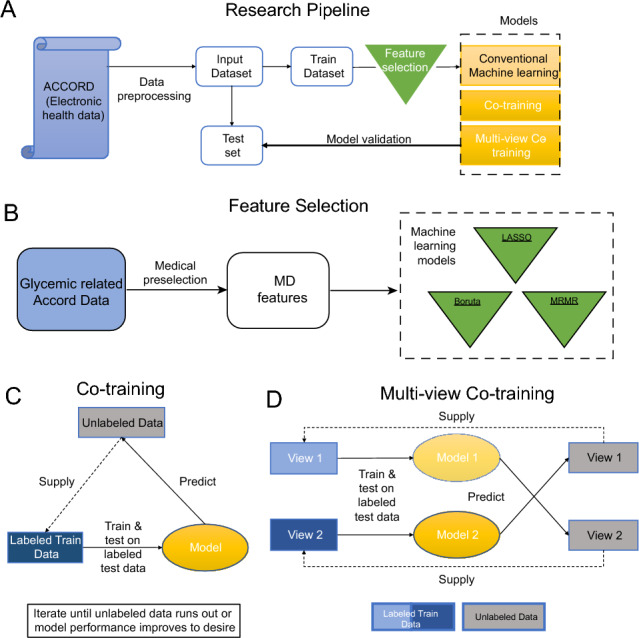
Research pipeline, feature selection methods and architecture of the models. **(A)** The overall structure of our research pipeline. **(B)** Diagram of the feature selection process. MD, LASSO, Boruta, and MRMR are feature selection algorithms. **(C)** Single-view co-training machine learning algorithm steps. **(D)** Multi-view co-training machine learning algorithm steps.

**Figure 2:**
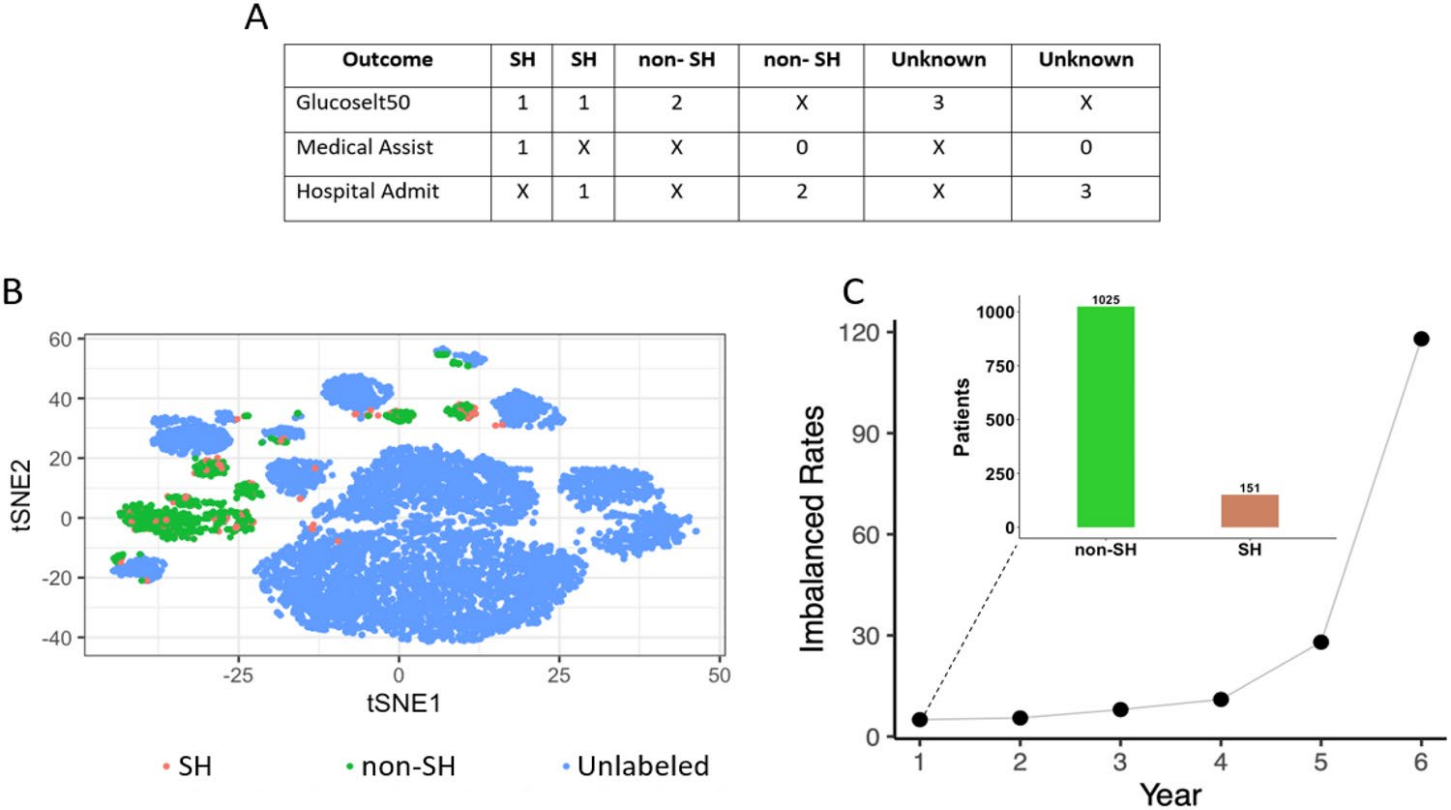
Visualization of the class of the dataset. **(A)** Related conditions for the dependent variable to be defined as SH. **(B)** Visualization of *t*-SNE of the high-dimensional dataset (17 dimensions) based on the ACCORD dataset colored by labeled (SH/non-SH) and unlabeled. Each dot represents a sample using MD. The labeled data is represented by green and red dots, corresponding to SH and non-SH events, respectively. Unlabeled data is represented by blue dots. **(C)** Hypoglycemia rates (non-SH/SH) of the dataset by year represented by solid circles. Class distribution of hypoglycemia events for the first year data represented by bar charts.

In this study, we use the data from ACCORD EHRs dataset and propose a multi-view co-training ML model as an effective semi-supervised learning (SSL) method for predicting SH events as illustrated in Fig. 1. The current study is the first to predict SH events, especially by proposing a multi-view co-training ML model. This choice is motivated not only by clinical needs to avoid hypoglycemia in the clinical setting but also by the unlabeled data available in the ACCORD dataset. In particular, we encountered the following two problems with the ACCORD dataset during our study: i) Imbalanced data. The dataset is highly imbalanced (see Fig. 2); while the imbalance rate is approximately 1:6.79 for the first year, this ratio increases to 1:120 by the end of the sixth year. ii) Features plurality. In managing and analyzing large datasets such as the ACCORD dataset utilized in this study, one encounters the challenge of a plurality of features. This refers to the extensive number of variables collected, which, while enriching the dataset, also complicates the analysis due to high dimensionality. High-dimensional data can lead to overfitting. This occurs when there is a high number of input features and the algorithm overly prioritizes maximizing the distinction between different classes^14^. To address these challenges, our study employs feature selection techniques and a multi-view co-training approach, allowing us to distill the most informative features and enhance the predictive accuracy of our models, particularly in the context of imbalanced datasets typical of medical data where events of interest are rare.

The primary objectives of this study are: (i) to develop a ML model for predicting long-term SH events in patients with T2DM, which will enable patients and healthcare providers to take appropriate precautions; and (ii) to identify the most effective features for predicting long-term SH events. Specifically, we are proposing a new multi-view co-training model for the long-term prediction, based on the ACCORD^15^ dataset. By achieving these goals, we hope to contribute to the development of more effective methods for managing SH events in patients with T2DM.

## Related work

   The application of ML techniques in EHRs data has gained significant attention in recent years^16–18^, with a specific focus on EHRs data for diabetes prediction and management^19–21^. Zheng et al.^20^ suggested a semi-automated ML model to distinguish between individuals with and without T2DM. They wanted to raise recall rates while keeping false positive rates at a minimum. To accomplish this, they performed feature engineering and then trained various conventional ML models, including k-nearest-neighbors, Naive Bayes (NB), decision tree, Random Forest (RF), support vector machine, and logistic regression, based on the selected features. Nguyen et al.^21^ developed a hybrid system to predict the onset of diabetes by combining wide and deep learning models using EHRs data. Their hybrid approach outperformed other models, achieving an accuracy level of 84.28.

The ACCORD EHRs dataset used in this study is characterized by a considerable number of features. Using this large dataset, clinical researchers focusing on diabetes could conduct risk prediction studies to identify factors that contribute to SH. In recent years, ML models have increasingly been applied to predict severe hypoglycemia in patients with diabetes. Ruan et al.^22^ analyzed data from 17,658 inpatients at a large university hospital, using various ML algorithms to predict hypoglycemia, with the XGBoost model achieving an impressive AUROC of 0.96. This significantly outperformed traditional logistic regression models, which had an AUROC of 0.75, highlighting the potential of advanced ML models to enhance predictive accuracy in clinical settings. Furthermore, Sudharsan et al.^23^ developed a probabilistic model trained on self-monitored blood glucose readings from real patients, achieving a sensitivity of 92% and a specificity of 70% for predicting hypoglycemic events within 24 hours. These findings suggest that incorporating routine clinical data, such as self-monitored blood glucose (SMBG) readings and medication schedules, into ML models can significantly enhance their predictive power. Supervised learning (SL) is one of the most widely used ML sub-fields in medical research, where labeled data is used in the training process to make decisions or predictions, such as in classification problems in pediatrics^24^, early diagnosis of cancer^25^, identification of drug candidates^26^, predicting 1-year cardiovascular events^27^ and predicting blood glucose levels in T2D^28^. Hong et al.^29^ developed a ML model using EHRs from over 1.4 million older adults, achieving an AUROC of 0.978 with XGBoost, which significantly outperformed traditional models in predicting SH requiring hospitalization. Yang et al.^30^ developed a hypoglycemia prediction model using XGBoost and natural language processing on EHR from 29,843 type 2 diabetes patients, achieving an AUC of 0.82 and accuracy of 0.93, thus demonstrating effective hypoglycemia risk prediction. Additionally, the field of hypoglycemia prediction using ML faces several challenges, including the variability in patient responses to treatments and the asymptomatic nature of many hypoglycemic events. Mujahid et al.^31^ provided a comprehensive review of the recent literature, highlighting the evolution from simple detection to more complex prediction algorithms that anticipate events well before they occur, thus allowing timely clinical interventions. While typical SL requires labeled data exclusively, it may not always be feasible in medical research due to limited availability or missing outcomes. SSL methods address this limitation by incorporating unlabeled data. SSL methods are another sub-field of ML that combines SL and unsupervised learning by using both labeled and unlabeled data together. The main purpose of SSL methods is to increase the performance of labeled data by using unlabeled observations or to increase the number of labels by producing pseudo labels. Due to these reasons, SSL models have been widely applied to medical studies by integrating labeled and unlabeled data, such as in cardiovascular risk prediction^32^, diabetes disease diagnosis^33^, microRNA prediction^34^, early prediction of pregnancy–associated hypertension^35^, and medical image analysis^36^.

In this research, we introduce 1-year SH prediction models that use both SSL and SL algorithms. Figure 1 provides a comprehensive visual representation of our research methodology, highlighting the key components and steps involved in our study. In Fig. 1, Panel (A) presents the overall pipeline structure, highlighting the various stages involved in our study. Panel (B) focuses on the feature selection process, illustrating the implementation of feature selection algorithms such as medical selection criteria (selected features by medical doctors), LASSO, Boruta, and MRMR. We refer to medically selected features as “MD” in this study. Panels (C) and (D) present the step-by-step processes of the single-view and multi-view co-training ML algorithms, respectively.

## Methods

### Objectives

The primary objective of this study is to develop a robust ML framework to accurately predict SH events in patients with T2DM. By utilizing a multi-view co-training approach on an imbalanced dataset, we aim to improve prediction accuracy by combining SSL and SL methods, leveraging both labeled and unlabeled data. Additionally, we seek to identify and utilize the most effective clinical and demographic features for predicting SH events, thus providing explainable artificial intelligence (XAI) insights and offering transparency and interpretability in the model’s predictions. The study also aims to compare the performance of the proposed multi-view co-training model with conventional single-view co-training models and other existing models, highlighting improvements in specificity, sensitivity, and overall accuracy. Ultimately, this research offers practical guidelines for clinicians on choosing between different models based on their priority for sensitivity or specificity in diagnosing SH, contributing to better patient management and early intervention strategies.

### Dataset description

*Study population* The design and outcomes of the ACCORD trial have been published before^37,38^. Data from 10,251 enrolled participants with clinical diagnoses of T2DM were collected through the ACCORD study. The study’s participants were mostly middle-aged and elderly patients ranging from 40 to 82, with an average of 62.2 years and an average diabetes duration of 10 years. Of the total participants, the majority were white (64.8%) and male (61.4%). In our study, after performing missing data imputation, we proceeded with the analysis using a total of 10,244 observations. Table 4 in supplementary material (SM) displays the mean ± standard deviations and percentages (%) for the selected variables in the ACCORD dataset.

*Outcome and predictors* We determined the response variable of the ACCORD dataset according to Fig. 2A. According to Fig. 2A, “Glucoselt50” is assigned as a value of 1 if the blood glucose level is below 50 mg/dl, 2 if it is above 50 mg/dl, and 3 if no information is available. “Medical Assist” is assigned 1, if medical assistance is required, 0 if not required. “Hospital Admit” is assigned a value of 1, if hospital admission is required, 2 if it is not required, and 3 if there is no information. Finally, the outcome is assigned as SH, non-SH, or Unknown based on the information provided by the patients. Patients who provided the following information were assigned as SH event; patients with a Blood Glucose level below 50 and either requiring Medical Assistance or Hospital Admission. For patients who provided the following information is assigned as non-SH; Blood Glucose is higher than 50. In addition, no medical assistance is required, and hospital admission is not needed assigned as value 0, indicating it is a non-SH event. Patients for whom we could not obtain information were assigned as Unknownt; if the Blood Glucose information is unknown or Medical Assistance is not required and unknown Hospital Admission. The ethics committee of Beth Israel Deaconess Medical Center determined that this study was exempt re: review and approval.

To create predictors, first, like Ma et al.’s study^39^, created all 116 candidate risk features listed in SM Table 3. After that, as represented in Fig. 1B, we created 116 relevant risk factors in the ACCORD dataset, then selected the top-12 risk factors. The medical estimators of the ACCORD dataset were chosen as follows: hemoglobin A1c (HbA1c), fasting plasma glucose (FPG), general health check (g1check), diabetes education (g1diabed), nutritional education (g1nutrit), sulfonylurea, meglitinide, NPH or L (NPHL) insulin, regular insulin (Reg Insulin), long-acting insulin (La Insulin), other bolus insulin (Othbol Insulin), and premixed insulin. As some of the features are longitudinal, we computed the mean and standard deviations of the observations and this process resulted in the following 17 variables: hba1c mean, hba1c std, fpg mean, fpg std, g1check mean, g1check std, g1diabed mean, g1diabed std, g1nutrit mean, g1nutrit std, sulfonylurea mean, meglitinide mean, nphl mean, reg insulin mean, la insulin mean, othbol insulin mean, premix insulin mean (see the SM Table 9). Additionally, the ACCORD dataset participants were followed for approximately 4 to 8 years^15^. We decided to work on the 2-year prediction, because the least imbalanced rate is seen for the first year.

*Unlabeled dataset* The ACCORD dataset contains 9068 unlabeled and 1176 labeled data. First, we started working with the labeled dataset, but we could not obtain significant results, and subsequently, we decided to include the unlabeled data in our analysis.

*Views* In our study, we propose a multi-view co-training ML model as SSL. To begin the analysis, we first started with three different views. These are, glycemic variables (View 1): FPG, HBA1C; glycemic management and medications (View 2): g1check, g1diabed, g1nutrit, sulfonylurea, meglitinide, NPHL insulin, reg insulin, la insulin, othbol insulin, and premix insulin; (View 3): years of diabetes, live alone, education level, body mass index (BMI), participant waist circumference (cm), race, age, and gender. We examined and compared these three views and ranked View 1 and View 2 as more effective. Therefore, we generated two views for classification, glycemic variables based (View 1) and glycemic management and medications based (View 2).

*Missing data imputation* In this study, we applied two different methods, namely the last-observation-carried-forward (LOCF)^40,41^, for the time-series observations, and the median imputation, for the non-time-series observations, to handle missing data.

*Feature selection and model validation* The feature selection algorithm is a method that helps to identify the most relevant variables from the input data and reduces it to a lower-dimensional dataset. Feature selection methods for classification tasks can be categorized into two groups^42^: expert knowledge-based feature selection methods, and automatic feature selection methods such as filter, wrapper, and embedded feature selection algorithms. In particular, we utilized the Boruta, MRMR, and LASSO methods as automatic feature selection algorithms. In the MRMR method, researchers should define the number of features to be selected in advance. Therefore, we needed to determine how many features should be selected by the MRMR method. To do this, MRMR selected features from 1 to 17, and then we calculated the AUC value for each. Finally, we obtained the highest AUC with four features, as shown in SM Fig. 2. Furthermore, we not only evaluate the individual performances of these three feature selection methods but also consider the features that are selected by all of them as effective features. We incorporated a technique into our analysis, namely the “consensus and majority vote feature selection” rule^43^, where the feature is considered an important feature if it is selected by all of the base feature selection methods in agreement. We have provided an explanation of the feature selection algorithms in the “Feature selection methods” section of the SM and listed all the expert knowledge-based selected features in Table 1.

**Table 1:** ACCORD dataset MD features selected by feature selection algorithms, including mean and standard deviation. Bold features are effectively selected by the consensus and majority vote rule.

MD features	Feature details	Selected by FS algorithms
**HbA1c**	Glycated hemoglobin (%), Continuous	LASSO, Boruta, MRMR
**FPG**	Fasting glucose (mg/dL), Continuous	LASSO, Boruta, MRMR
g1check	Blood sugar check frequency, Continuous	LASSO, Boruta
**g1diabed**	Diabetes education, Continuous	LASSO, Boruta, MRMR
g1nutrit	Nutrition education, Continuous	LASSO, Boruta
Sulfonylureas	Categorical	LASSO
Meglitinides	Categorical	
**Nph/L insulin**	NPH/L Insulins, Categorical	LASSO, Boruta, MRMR
Regular insulins	Categorical	
LA insulin	Lispro/Aspart insulins, Categorical	
Othbol insulin	Other Bolus Insulins, Categorical	LASSO, Boruta
Premixed insulin	Categorical	LASSO

### Proposed methodology

*Classification pipeline* All classifiers in this study are created using the caret package^44^ in R programming language 4.1.3. We start by employing conventional ML algorithms to process the entire labeled dataset. Across the entire study, the dataset is split into two sets (see Fig. 1A): A 20% sample of the data is used to test the classifier’s performance, while the remaining 80% is used to train the classifier. We built models using the training dataset and tested their performance with 5-fold cross-validation. We first assessed the performance of several distinct classifiers on the ACCORD dataset by calculating the classification accuracy. Specifically, we only used conventional machine learning methods, including Logistic Regression (LR), XGBoost, NB, Support Vector Machine (SVM), and Random Forest (RF). Then, we further evaluated the performance of single-view co-training and multi-view co-training models with NB and RF models; Naive Bayes classifier: A classification algorithm that operates on Bayes’ theorem and involves probabilities. Random forest: An ensemble classification or regression method that uses the decision tree algorithms^45^.

*Single-view co-training model* It is obvious in Fig. 1C that labeled data is split as train and test set, and the model is initially trained (Step 1). Afterward, the trained model is used to estimate the unlabeled data (Step 2), and the most confident pseudo-labels are selected by the probability $\Theta$ higher than 0.90. In the next step (Step 3), the pseudo-labeled data and labeled data are concatenated. The model then makes predictions on unseen test data (Step 4), and finally, the results are evaluated (Step 5). Step 1 and Step 5 are repeated until new unlabeled data can no longer be added. We also tested the heterogeneity of the data by applying the cross-validation method. We showed the mean accuracy metrics of test results for each iteration in single-view co-training for NB using MD features in SM Figure 9.

*Multi-view co-training model* Blum^46^ introduced the co-training algorithm, which is a SSL algorithm, and numerous studies have been conducted on this topic^46–49^. The multi-view co-training method utilizes both views in tandem to supplement a much smaller number of labeled examples with unlabeled data. Blum^46^ first defined the labeled (*L*), unlabeled dataset (*U*) and unlabeled pools ($U'$) (created for each View 1 and View 2), and set the number of iteration *k*, then divided the input space $X = X_1 \times X_2$, so that $X_1$ and $X_2$ corresponding to two distinct sufficient and redundant views (View1 and View2) of the *X*, and they trained each view from the labeled data (*L*) by $h_1$ and $h_2$ classifiers. Then, the co-training method allows $h_1$ to label *p* positive and *n* negative most confident labels from the unlabeled ($U'$) set (for View 2) as a pseudo label and again $h_2$ to label *p* positive and *n* negative most confident labels from the unlabeled ($U'$) set (for View 1), so this prevents from over-training. Finally, the algorithm adds these confident labels to *L* and deletes the selected confidence labels from $U'$ (see Fig. 1D). Thus, the multi-view co-training method allows learning from both a few labeled and unlabeled data.

*Combining the views* Instead of performing classification, multi-view co-training is typically utilized to generate larger labeled data. In order to use it as a classification tool, it is necessary to combine the views generated at the end of the iteration within the multi-view co-training. There are many methods to combine the views, but we prefer the naive AND and OR rule to combine final predictions coming from View 1 and View 2. The AND rule assigns the result as 1, if the results from the *i*th observation of both views are 1. The OR rule assigns the result as 1, if only one of the results from the *i*th observation from both views is 1. *Multi-view co-training algorithm steps*

The proposed algorithm is a Multi-View Co-Training Machine Learning Model designed to predict severe hypoglycemia (SH) in patients with Type 2 Diabetes Mellitus (T2DM). This approach leverages both labeled and unlabeled data, integrating semi-supervised learning (SSL) and supervised learning (SL) techniques to enhance prediction accuracy, especially in imbalanced datasets.

The proposed algorithm is a Multi-View Co-Training Machine Learning Model designed to predict severe hypoglycemia (SH) in patients with Type 2 Diabetes Mellitus (T2DM). This approach leverages both labeled and unlabeled data, integrating semi-supervised learning (SSL) and supervised learning (SL) techniques to enhance prediction accuracy, especially in imbalanced datasets.

### Algorithm

The proposed algorithm is a Multi-View Co-Training Machine Learning Model designed to predict SH in patients with T2DM. This approach leverages both labeled and unlabeled data, integrating SSL and SL techniques to enhance prediction accuracy, especially in imbalanced datasets. We show the algorithm steps below:

- Data preprocessing: Apply preprocessing steps as explained in the “Method” section.
- Feature selection methods: Apply multiple feature selection techniques.
  - MD (Medical Selection Criteria): Based on clinical relevance and expert knowledge.
  - LASSO (Least Absolute Shrinkage and Selection Operator): Reduces the dimensionality by penalizing the absolute size of coefficients.
  - Boruta: A wrapper algorithm that selects all relevant features by comparing the importance of real features to shadow features.
  - MRMR (Minimum Redundancy Maximum Relevance): Selects features that are most relevant to the target variable while ensuring minimal redundancy among them.
- Algorithm:
  - Input Dataset: ACCORD EHRs dataset.
  - Views: Create two distinct views for co-training:
    * View 1: Glycemic variables.
    * View 2: Glycemic management and medications.
  - Multi-View Co-Training Procedure:
    * Initialization:
      - Split the labeled dataset into training and test sets (80% training, 20% testing).
      - Initialize two models, one for each view.
    * Iteration Process:
      - Train each model on its respective view using the labeled data.
      - Use each trained model to label the most confident unlabeled data points (pseudo-labeling).
      - Add these pseudo-labeled points to the training set.
      - Repeat the process until constraints are reached.
    * Combination of Views:
      - AND Rule: A data point is classified as positive only if both views agree.
      - OR Rule: A data point is classified as positive if at least one view predicts a positive outcome.
- Model Evaluation:
  - Assess model performance using metrics such as Accuracy, Specificity, Sensitivity, Positive Predictive Value (PPV), and Negative Predictive Value (NPV).
  - Perform cross-validation to ensure robustness and avoid overfitting.

## Results

### Data quality checking

Upon completing the labeling process based on Fig. 2A, we obtained a total of 1,176 labeled data points, consisting of 151 cases of SH and 1,025 non-SH cases, along with 9,068 unlabeled data points. Figure 2B is a projection of the data obtained using the t-distributed stochastic neighbor embedding (*t*-SNE) method^50^, as we revealed by the *t*-SNE, there is no clear separation based on the target classes of SH non-SH in labeled data. For this reason, we thought that we could obtain meaningful information from the unlabeled data. In addition, the SH rates of these patients for 6 years are shown in Fig. 2C for each year.

In the forthcoming subsections, we first discussed the results obtained from conventional machine learning models in the *“*Conventional machine learning results” section. This was followed by insights from the single-view co-training model in the *Results Based on the Single-View Co-Training Model* Subsequently, we delved into the results based on the multi-view co-training model in the *Results Based on the Multi-View Co-Training Model* Finally, we synthesized all findings under the section “Combining the views of the multi-view co-training model” where we integrated information from different views to enhance prediction accuracy and model robustness.

*Conventional machine learning results* We first analyzed and compared various feature selection algorithms on a labeled dataset. First, to prevent overfitting and informed by clinical knowledge on SH, we performed our analysis using a total of 17 features. Consequently, we performed feature selection with four different algorithms, MD, LASSO^51^, Boruta^52^ and MRMR^53,54^, as shown in Fig. 1B. Following the feature selection process, we compared the performance of representative ML algorithms. In the testing data, as seen in Table 2, the NB model with MRMR feature selection shows the best performance among all classifiers, with specificity and accuracy of 0.740 and 0.696, respectively. Also, the NB model with LASSO feature selection demonstrates the highest performance for NPV and sensitivity of 0.942, and 0.986, respectively. Lastly, the RF model with MD achieves the highest performance with a positive predictive value (PPV) of 0.194 and an F1-score of 0.303. Additionally, the 5-fold AUC-ROC curves of the conventional ML algorithms can be viewed in SM Figure 8. We have provided an explanation of the formula for performance measures in the “Performance measures” section of the SM.

**Table 2:** Comparison of results from conventional models using MD, LASSO, Boruta, and MRMR feature selection algorithms. NPV: Negative predictive value; PPV: Positive predictive value; Spec: Specificity; Sens: Sensitivity; Acc: Accuracy; F1: F1-score. In terms of predictive performance, the NB model outperforms all other models. Each accuracy metric’s highest value is bold.

Feature selection	Algorithms	NPV	PPV	Spec	Sens	Acc	F1
MD	LR	0.913	0.184	0.591	0.612	0.594	0.280
MD	XGBoost	0.907	0.170	0.560	0.603	0.565	0.263
MD	NB	0.924	0.131	0.038	0.979	0.159	0.230
MD	SVM	0.912	0.188	0.617	0.597	0.614	0.285
MD	RF	0.927	**0.194**	0.572	0.697	0.588	**0.303**
LASSO	LR	0.906	0.180	0.611	0.567	0.605	0.271
LASSO	XGBoost	0.909	0.172	0.559	0.617	0.566	0.267
LASSO	NB	**0.942**	0.130	0.026	**0.986**	0.150	0.229
LASSO	SVM	0.911	0.192	0.629	0.583	0.622	0.286
LASSO	RF	0.924	0.191	0.578	0.681	0.590	0.297
Boruta	LR	0.909	0.181	0.597	0.594	0.597	0.276
Boruta	XGBoost	0.908	0.168	0.543	0.627	0.553	0.264
Boruta	NB	0.938	0.130	0.027	**0.986**	0.150	0.230
Boruta	SVM	0.912	0.187	0.608	0.601	0.607	0.284
Boruta	RF	0.927	0.192	0.568	0.699	0.584	0.301
MRMR	LR	0.910	0.184	0.623	0.574	0.617	0.277
MRMR	XGBoost	0.906	0.166	0.552	0.610	0.559	0.259
MRMR	NB	0.894	0.184	**0.740**	0.407	**0.696**	0.248
MRMR	SVM	0.910	0.181	0.606	0.586	0.604	0.276
MRMR	RF	0.917	0.180	0.559	0.657	0.571	0.282

*Results based on the single-view co-training model* Despite the success of conventional models in demonstrating good performance in NPV, these models have shown limitations in other metrics that are crucial for practical implementation. Inspired by SSL, where integrating unlabeled datasets increases the performance of ML algorithms, we aim to incorporate a multitude of unlabeled observations in ACCORD data that remain to be explored. Due to limitations of space, we include the results for the single-view co-training model in the SM Table 1. As the single-view co-training method was not effective enough in improving conventional ML results, we switched to a different and more effective SSL method, the multi-view co-training method, as we discuss next. In addition, the confusion matrix of the single-view co-training results is listed in SM Figure 6.

*Results based on the multi-view co-training model* We fixed the number of iterations at 30, as experimented by Blum^46^. However, we executed different ratios of positive and negative pseudo-labels for selection, and we list the 5 negative/1 positive selection results in Table 3. In Table 3, we compare the performance of our multi-view co-training model on datasets processed using MD and MRMR feature selection methods. The model employed NB and RF algorithms to handle the imbalanced data through under-sampling, enhancing predictive accuracy. The percentage improvement is calculated using the formula: $\text {Percentage} = \frac{(\text {ViewX.last} - \text {ViewX.1st}) \times 100}{\text {ViewX.1st}}$ where “ViewX.last” and “ViewX.1st” represent the final and initial accuracies, respectively, within each view. To provide a clear understanding of the co-training steps and algorithmic configurations, detailed explanations and pseudo-code are available in the SM (Section “Algorithmic representation of the multi-view co-training model”). This includes how each view was processed, the rationale behind the selection of algorithms, and the under-sampling strategy employed to manage data imbalance. The effectiveness of the selected features and their impact on model performance were assessed using accuracy measures such as precision, recall, and F1 score, and analyses were conducted using R-programming language, ensuring reproducibility of our results. Table 3 shows the accuracy measures for the first and the last iterations, as well as the percentage of gain or loss for each model and view. Table 3 indicates that in View 2, the highest contribution rates are observed for specificity (144.732%) and accuracy (90.416%) in the RF MRMR feature selection model. Additionally, performance curves of the accuracy, negative predicted value (NPV), and AUC-ROC results are presented in SM Figure 3. Moreover, we provide the confusion matrix of the multi-view co-training model results in SM Figure 7.

**Table 3:** Comparing multi-view co-training model results on MD and MRMR selected data with an under-sampling imbalanced solution. Results of the MD data and MRMR data. Percentage=(ViewX.last-ViewX.1st)$\times$100/ViewX.1st. Both NB and RF achieve great improvements using these two selected features.

	MD						MRMR					
	NPV	PPV	Spec	Sens	Acc	F1	NPV	PPV	Spec	Sens	Acc	F1
NB												
View1.1st	0.883	0.168	0.775	0.311	0.714	0.217	0.887	0.192	0.804	0.314	0.740	0.233
View1.last	0.884	0.204	0.867	0.230	0.785	0.212	0.883	0.213	0.874	0.224	0.790	0.214
Percentage	0.075%	21.238%	11.971%	− 25.943%	9.878%	− 2.180%	− 0.457%	10.870%	8.674%	− 28.643%	6.785%	− 8.149%
View2.1st	0.905	0.157	0.485	0.649	0.506	0.252	0.910	0.172	0.570	0.609	0.575	0.267
View2.last	0.876	0.149	0.842	0.190	0.758	0.155	0.876	0.176	0.876	0.155	0.784	0.155
Percentage	− 3.174%	− 4.844%	73.755%	− 70.640%	49.759%	− 38.270%	− 3.720%	2.066%	53.583%	− 74.495%	36.396%	− 41.921%

RF												
View1.1st	0.898	0.162	0.564	0.568	0.564	0.251	0.897	0.157	0.544	0.579	0.548	0.246
View1.last	0.891	0.183	0.757	0.368	0.708	0.244	0.884	0.161	0.747	0.333	0.693	0.216
Percentage	− 0.789%	12.805%	34.099%	− 35.115%	25.491%	− 2.853%	− 1.502%	2.817%	37.150%	− 42.596%	26.345%	− 12.420%
View2.1st	0.906	0.163	0.528	0.624	0.541	0.258	0.931	0.161	0.393	0.785	0.444	0.266
View2.last	0.886	0.177	0.789	0.308	0.728	0.224	0.874	0.221	0.961	0.065	0.845	0.136
Percentage	− 2.273%	8.279%	49.543%	− 50.626%	34.592%	− 13.252%	− 6.130%	37.319%	144.732%	− 91.745%	90.416%	− 49.023%

*Combining the views of multi-view co-training model* Up to now, we have assessed the performance of each view created through the multi-view co-training model. Subsequently, we interpret the results of combining the information obtained from these views. Multi-view co-training is often used to increase the number of labeled data, but to employ multi-view co-training as a classification method, we need to combine the outputs. We employ a naive approach where the results are combined using both AND and OR rules. AND and OR rule results can be seen in SM Table 2 and the comparison of these combined results with other findings is presented in Table 4, showing the top-performing outcomes. We list the 5 negative/1 positive selection results in SM Figure 1, while the other results (3 negative and 1 positive, and 7 negative and 1 positive) can be found in the SM Tables 5-8 and in SM Figure 4-5. Accordingly, we note that the AND rule produces better results for specificity, accuracy, and PPV measures, with values of 0.993, 0.868, and 0.300, respectively. On the other hand, the OR rule outperforms in terms of NPV, sensitivity, and F1, with values of 0.906, 0.589, and 0.267, respectively. (see more details in SM).

*Comparison with existing best models* Finally, we can compare the top-performing results from conventional, single-view co-training, and multi-view co-training ML algorithms in Table 4. The multi-view co-training approach employs two rules for prediction: the AND Rule assigns a positive label “1” only if both views agree on a positive prediction, and the OR Rule assigns a positive label if at least one of the views predicts positive. These rules were applied after the last iteration of the multi-view training process, where each model’s predictions were independently validated against the test data. It is seen in Table 4 that the conventional models are only successful in predicting the majority class (non-SH) events, with NPV at 0.942 and sensitivity at 0.986, respectively. On the other hand, the multi-view models demonstrate a higher success rate in predicting SH events, achieving the best result for PPV at 0.300, specificity at 0.993, and accuracy at 0.868, respectively.Additionally, we show the performance comparison between single-view co-training and multi-view co-training ML model results in SM Figure 10.

**Table 4:** Performance comparison with existing best models. Each accuracy metric’s highest value is bold. Multi-view co-training performs the best, but only the conventional NB LASSO meets the clinical criteria of high sensitivity.

Method	NPV	PPV	Spec	Sens	Acc	F1
Conventional NB LASSO	**0.942**	0.130	0.026	**0.986**	0.150	0.229
Conventional RF MD	0.927	0.194	0.572	0.697	0.588	**0.303**
Single-view co-training RF MD	0.924	0.186	0.561	0.689	0.577	0.293
Multi-view co-training RF MD-OR Rule	0.906	0.173	0.584	0.589	0.585	0.267
Multi-view co-training RF MRMR-AND Rule	0.873	**0.300**	**0.993**	0.027	**0.868**	0.037

## Discussion

   In this study, ML models were developed to predict the occurrence of SH events and identify the effective features that contribute to such predictions for patients with T2DM in the ACCORD data. The key findings of the study are: (1) top-12 features were selected by expert knowledge-based selection for predicting SH events; (2) 1-year SH prediction models were developed using both SSL with a multi-view co-training method and SL with RF or NB models; and (3) the most effective features were proposed based on both expert knowledge-based selection and automatic feature selection methods for predicting SH events. (4) a shiny app^55^ was designed to facilitate further analysis by researchers using the proposed multi-view co-training ML method.

The findings indicate that the suggested multi-view co-training approach exhibits superior performance in attaining elevated levels of specificity, PPV, and overall accuracy. However, conventional ML algorithms surpass it in terms of sensitivity, NPV, and F1 measures when predicting SH. Throughout this study, we employed different feature selection techniques prior to constructing the predictive model, and we explored the most suitable methods from both automatic and expert knowledge-based feature selection approaches. Specifically, we applied Boruta, MRMR, and LASSO methods for automatic feature selection algorithms, while the expert knowledge-based method used in the study was referred to as the MD method. We achieved better results in predicting SH when we used automatic feature selection methods rather than the merely MD method. Among them, we learned that the MRMR feature selection algorithm was the most effective at identifying the optimal features for the multi-view co-training model. In addition, we identified the effective features for the 1-year SH prediction that are the common selection of these four features using the “consensus and majority vote feature selection” rule^43^; fast plasma glucose (FPG), hemoglobin A1c (HbA1c), general diabetes education (other than nutrition) (g1diabed) and NPH or L (NPHL insulin) insulins. Our framework also provides XAI^56,57^ by identifying key predictors for hypoglycemia. Notably, HbA1c, FPG and NPHL insulins are beyond the control of patients and medical doctors, but general diabetes education can be controlled, and the development of effective self-management skills in patients has the potential to lower the incidence of hypoglycemia and increase knowledge regarding hypoglycemia^58^. Additionally, there is a risk between HbA1c^58–62^ or FPG^59,60,62^ and a higher risk of hypoglycemia. Finally, the most promising results are obtained by implementing the RF multi-view co-training method with the MRMR feature selection using the AND rule; NPV: 0.873, PPV: 0.300, specificity: 0.993, and accuracy: 0.868 and the conventional NB model with LASSO feature selection; NPV:0.942, and sensitivity: 0.986. The dataset is imbalanced and accuracy can vary significantly in imbalanced datasets^63^, and the results should be interpreted accordingly. The proposed multi-view co-training method achieved two to eight times higher performance measurements in metrics such as PPV, specificity, and accuracy compared to the best supervised ML methods. Therefore, it can be said that the proposed method is more effective than supervised methods. Additionally, multiple ML approaches were considered in this study, in order to determine the “best” modeling technique. However, it is crucial to know that there is not just one ideal model. For example, if the goal is to avoid SH, then “best” might be the model with the highest sensitivity ^64^, or high specificity would be “best” if the goal is to avoid unnecessary treatments or interventions. Therefore, we recommend the conventional NB LASSO to avoid SH, and the multi-view co-training RF MRMR-AND rule to avoid unnecessary intervention. Alternatively or additionally, more frequent medical monitoring and/or more advanced medications, mainly those not leading to hypoglycemia such as SGLT2is or GLP-1 analogues, would be needed for subjects who would qualify as high risk based on these models.

## Conclusion and future works

   We proposed ML methods to predict SH events in T2DM patients, systematically examining our approaches using a 7-year clinical trial, namely ACCORD. Our study employed SSL, specifically a novel multi-view co-training method, alongside SL methods such as RF and NB, to enhance predictive accuracy. We have achieved very high sensitivity in models conventional NB LASSO (close to 99%) and thus we suggest that doctors or researchers prioritizing high sensitivity (usually the first step in the diagnostic process) might prefer conventional methods like NBs, while those interested in high specificity (usually as a second step in the diagnostic process) could explore Multi-view Co-training methods. We also identified four key features highly effective in SH prediction: fasting plasma glucose (FPG), hemoglobin A1c (HbA1c), general diabetes education (g1diabed), and use of NPH or L Insulins (NPHL insulin).

This study also offers explainable artificial intelligence (XAI) insights, revealing the factors influencing predictions and enhancing transparency. The multi-view co-training approach significantly improved the PPV, specificity, and accuracy of SH event predictions. It effectively utilizes data with missing labels, which could greatly benefit the T2DM patient population by improving prediction accuracy and ultimately leading to more personalized treatment plans and enhanced health management. Additionally, the identified predictive features can aid clinicians in making better decisions.

We have developed a user-friendly Shiny app^55^ to facilitate the use of the multi-view co-training method, hosted at https://datascicence.shinyapps.io/MultiViewCoTraining/. This tool represents a significant advancement in SSL, providing a platform for researchers and practitioners to explore and compare Self-Training and Co-Training methodologies. Users can upload their data, adjust parameters such as iteration count and train/test split ratio, and visually compare the outcomes of different SSL strategies. This enhances the accessibility of these methods and encourages a deeper understanding of their practical implications, particularly in scenarios where labeled data is scarce or costly to obtain.

In conclusion, our 1-year SH prediction models, based on data from over 10,000 T2DM patients, demonstrate that our multi-view co-training method, incorporating RF, has improved specificity. Future enhancements could include integrating Deep Belief Networks as suggested by Reddy et al.^65,66^, to further boost the accuracy and robustness of our predictions, offering promising directions for optimizing patient management and resource allocation. The ability to predict SH over a year allows for early and proactive management, significantly enhancing patient care by providing healthcare professionals with ample time to adjust treatments and implement preventive measures. Integration with continuous glucose monitoring systems could further enhance this model’s clinical utility, offering real-time adjustments and improving patient safety.

## Supplementary Information

Supplementary Information.

## Data Availability

The Action to Control Cardiovascular Risk in Diabetes (ACCORD) data is available upon request from the National Heart, Lung, and Blood Institute (NHLBI). https://biolincc.nhlbi.nih.gov/studies/accord/

## Code availability

All codes are available on GitHub at https://github.com/melihagraz/multiview_learning_R/tree/updated
