# Supplementary material for: Enhancing severe hypoglycemia prediction in type 2 diabetes mellitus through multi-view co-training machine learning model for imbalanced dataset
Source: Sci Rep. 2024 Sep 30;14:22741. doi: 10.1038/s41598-024-69844-z (PMC11444036; doi:10.1038/s41598-024-69844-z)
Supplement: Supplementary file 1 — Supplementary Information. [file 41598_2024_69844_MOESM1_ESM.pdf]

Enhancing Severe Hypoglycemia Prediction in Type 2 Diabetes Mellitus Through Multi-View Co-Training Machine Learning Model for Imbalanced Dataset

Melih Agraz[1,2,6*], Yixiang Deng[4,5*], George Em Karniadakis[1,3,†], and Christos Socrates Mantzoros[6,†,‡]

[1]Division of Applied Mathematics, Brown University, Providence, RI 02912, USA
[2]Department of Statistics, Giresun University, Giresun, 28200, Turkey
[3]School of Engineering, Brown University, Providence, RI 02912, USA
[4]Department of Biological Engineering, Massachusetts Institute of Technology, Cambridge, MA 02142, USA
[5]Ragon Institute of Mass General, MIT and Harvard, Cambridge, MA 02142, USA
[6]Department of Endocrinology, Beth Israel Deaconess Medical Center, Harvard Medical School, Boston, MA 02215, USA
[*]Contributed equally
[†]e-mail: george_karniadakis@brown.edu; cmantzor@bidmc.harvard.edu
[‡]Corresponding author

Supplementary Material (SM)

Candidate Risk Factors

Table 1: Comparison of the results obtained from a single-view co-training model using MD and MRMR feature selection data. Features of the MD data; FPG mean, FPG STD, HbA1c mean, HbA1c STD, g1check mean, g1check STD, g1diabed mean, g1diabed STD, g1nutrit mean, g1nutrit STD. Features of the MRMR data; FPG STD, HbA1c mean, g1diabed STD and NPHL insulin mean. Percentage=(X last iteration-X $1st$ iteration)×100/ (X $1st$ iteration). Based on the single-view co-training algorithm, we observe great improvement for the NB method in specificity, sensitivity, and accuracy.

	NB					
	NPV	PPV	Spec	Sens	Acc	F1
MD $1st$ iteration	0.924	0.131	0.038	0.979	0.159	0.230
MD last iteration	0.900	0.127	0.133	0.865	0.228	0.221
Percentage change	-2.60%	-2.63%	**249.21%**	-11.64	**43.38%**	-3.86
MRMR $1st$ iteration	0.894	0.183	0.740	0.407	0.696	0.248
MRMR last iteration	0.868	0.129	0.265	0.740	0.326	0.219
Percentage change	-2.89%	-29.94%	-64.17%	**81.76%**	-53.17%	-11.97%

	RF					
	NPV	PPV	Spec	Sens	Acc	F1
MD $1st$ iteration	0.920	0.185	0.570	0.663	0.582	0.289
MD last iteration	0.924	0.186	0.561	0.689	0.577	0.293
Percentage change	(0.40%)	(0.85%)	-1.52%	**3.81%**	-0.87%	**1.39%**
MRMR $1st$ iteration	0.916	0.176	0.548	0.658	0.562	0.277
MRMR last iteration	0.914	0.171	0.545	0.639	0.557	0.268
Percentage change	-0.20%	-3.09%	-0.72%	-2.92%	-0.89%	-3.2%

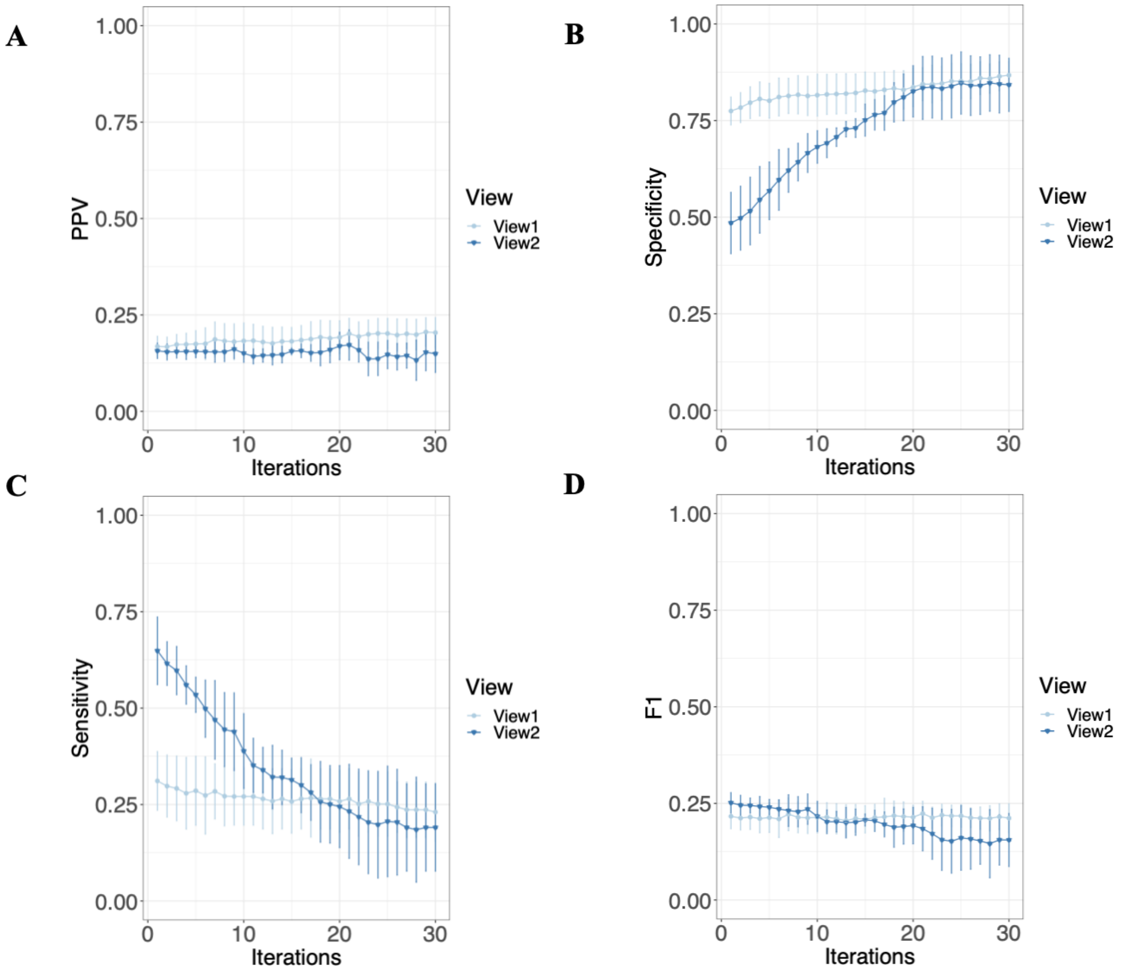

Figure 1: **Mean accuracy metrics of test results for each iteration in multi-view co-training for NB using MD features.** We split the data into two different labeled data sets View 1 and View 2. From the unlabeled data, we create two pools of datasets: U1 and U2. Following the training process, predictions are generated for both U1 and U2, and pseudo labels are created. The most confident labels are then added to the labeled dataset. The iteration is repeated thirty times. We observe a great improvement in specificity at the expense of accuracy and sensitivity. **(A)** Positive Predicted Value (PPV) or Precision **(B)** Specificity **(C)** Sensitivity **(D)** F1-score (F1).

Table 2: Results of combining views using both AND and OR rules. When we combine them in either way, RF wins, but it prevails in MD selection OR rule, while MRMR prevails in the AND rule. The AND rule yields higher PPV, specificity, and accuracy, whereas the OR rule yields higher NPV, sensitivity, and F1. More balanced specificity and sensitivity results are obtained with the OR rule.

AND Rule

	NPV	PPV	Spec	Sens	Acc	F1
NB-MD	0.870	0.050	0.982	0.007	0.856	0.012
NB-MRMR	0.872	0.167	0.981	0.026	0.858	0.046
RF-MD	0.877	0.245	0.961	0.086	0.849	0.128
RF-MRMR	0.873	**0.300**	**0.993**	0.027	**0.868**	0.037

OR Rule

	NPV	PPV	Spec	Sens	Acc	F1
NB-MD	0.892	0.179	0.728	0.404	0.686	0.248
NB-MRMR	0.889	0.183	0.770	0.351	0.716	0.241
RF-MD	**0.906**	0.173	0.584	**0.589**	0.585	**0.267**
RF-MRMR	0.885	0.161	0.715	0.371	0.671	0.224

Table 3: All candidate risk factors with folder names.

	**Variables**	**Description**	**Type**
1	Female	Gender: 0=Male, 1=Female	Num
2	Baseline age	Age (yrs): Randomization	Num
3	Arm	Randomization Arm: 1-8	Char
4	cvd_hx_baseline	CVD History at Baseline: 0=No, 1=Yes	Num
5	Raceclass	Race Class: White, Black, Hispanic, Other	Char
		Data Set Name: accord_key.sas7bdat	
6	sbp	Systolic Blood Pressure (mmHg)	Num
7	dbp	Diastolic Blood Pressure (mmHg) every month in the first four months; after that every two months	Num
		Data Set Name: bloodpressure.sas7bdat	
8	loop	Loop diuretics	Num
9	thiazide	Thiazide diuretics	Num
10	ksparing	Ksparing diuretics	Num
11	potassium	Potassium supplements	Num
12	a2rb	Angiotensin type 2 antagonists (ARB)	Num
13	acei	Ace inhibitors	Num

Continued on next page

Table 3 – continued from previous page

	Variables	Description	Type
14	dhp_ccb	Dihydropyridine calcium channel blockers	Num
15	nondhp_ccb	Non-dihydropyridine calcium channel blockers	Num
16	alpha_blocker	Peripheral alpha-blockers	Num
17	central_agent	Central alpha-adrenergic agonists	Num
18	beta_blocker	Beta-blockers	Num
19	vasodilator	Vasodilators	Num
20	reserpine	Reserpine	Num
21	other_bpmed	Other antihypertensive agents	Num
22	digitalis	Digitalis preparations	Num
23	antiarrhythmic	Anti-arrhythmics	Num
24	nitrate	Nitrates	Num
25	other_cvmed	Other cardiovascular drugs	Num
26	sulfonylurea	Sulfonylureas	Num
27	biguanide	Biguanides	Num
28	meglitinide	Meglitinides	Num
29	ag_inhibitor	Alpha-glucosidase inhibitors	Num
30	nphl_insulin	NPH or L Insulins	Num
31	tzd	Thiazolidinediones	Num
32	reg_insulin	Regular Insulins	Num
33	la_insulin	Lispro or Aspart Insulins	Num
34	othbol_insulin	Other Bolus Insulins	Num
35	premix_insulin	Premixed Insulins	Num
36	other_diabmed	Other Diabetes Treatments	Num
37	bile_sequestrant	Bile-acid sequestrants	Num
38	statin	HMG CoA reducatce inhibitors (statins)	Num
39	fibrate	Fibrates	Num
40	other_lipidmed	Miscellaneous lipid-lowering drugs	Num
41	cholest_abi	Cholesterol absorption inhibitors	Num
42	niacin	Niacin and nicotinic acid	Num
43	anti_coag	Oral anticoagulants (warfarin, coumadin, anisindione)	Num
44	anti_inflam	Non-steroidal anti-inflammatory agents (excluding aspirin)	Num
45	platelet_agi	Inhibitors of platelet aggregation (except aspirin)	Num
46	cox2	Cox-2 inhibitors	Num
47	aspirin	Aspirin	Num
48	thyroid	Thyroid agents	Num
49	progestin	Progestins	Num
50	estrogen	Estrogens (excluding vaginal creams)	Num
51	oral_asthma	Oral asthma drugs (except steroids)	Num
52	anti_depress	Any antidepressant	Num

Continued on next page

Table 3 – continued from previous page

	Variables	Description	Type
53	inhaled_asthma	Inhaled steroids for asthma	Num
54	oral_steroid	Oral steroids	Num
55	anti_psych	Any antipsychotic	Num
56	osteoporosis	Drugs for osteoporosis	Num
57	fluid_retention	Diuretic for fluid retention	Num
58	other_med	Any other prescribed medication	Num
59	vitamin	Vitamins and/or nutritional supplements	Num
60	otc	Over-the-counter medications	Num
		Data Set Name: concomitantmeds.sas7bdat	
61	Hba1c	glycosylated hemoglobin (%)	Num
		Data Set Name: hba1.sas7bdat	
62	chol	Total Cholesterol (mg/dL)	Num
63	trig	Triglycerides (mg/dL)	Num
64	hdl	High density lipoprotein (mg/dL)	Num
		Data Set Name: lipids.sas7bdat	
65	Total_Brain_Volume_ICV	Intracranial Volume (cc, algorithm)	Num
66	Gray_Matter_total_sum	Gray Matter, Total (cc, summed)	Num
67	white_matter_total_sum	White Matter, Total (cc, summed)	Num
		Data Set Name: mind_mri.sas7bdat	
68	FPG	Fasting plasma glucose (mg/dL)	Num
69	Serum creatinine	Serum creatinine (mg/dL)	Num
70	eGFR	eGFR from 4 variable MDRD equation (ml/min/1.73 m^2)	Num
71	UALB	Urinary albumin (mg/dL)	Num
72	UACR	Urinary albumin to creatinine ratio (mg/g)	Num
		Data Set Name: otherlabs.sas7bdat	
73	livealon	participant lives with one or more adults	Num
74	edu	participant's highest level of education	Num
75	yrsdiab	year of diabetes diagnosis	Num
76	yrslipi	year of hyperlipidemia diagnosis	Num
77	yrstens	year of hypertension diagnosis	Num
78	ulcer	foot ulcer requiring antibiotics	Num
79	protein	protein in urine	Num
80	hartfail	heart failure/CHF	Num
81	neuropat	neuropathy/nerve problems	Num
82	depressn	depression	Num
83	histhart	family history of heart disease, heart attack, or stroke	Num

Continued on next page

Table 3 – continued from previous page

	Variables	Description	Type
84	cigarett	smoked cigarettes in last 30 days	Num
85	smokelif	smoked more than 100 cigarettes during lifetime	Num
86	alcohol	number of alcoholic drinks consumed weekly	Num
87	waist_cm	waist circumference (cm)	Num
88	scrright	visual acuity score, right eye	Num
89	scrleft	visual acuity score, left eye	Num
90	retpathy	participant had retinopathy	Num
91	visloss	vision loss	Num
92	fankle	ankle reflexes	Num
93	BMI	Body mass index (kg/m^2)	Num
		Data Set Name: f07_baselinehistoryphysicalexam.sas7bdat	
94	g1nutrit	nutrition education, time in minutes	Num
95	g1diabed	general diabetes education (other than nutrition), time in minutes	Num
96	g1check	average frequency of blood sugar check, times per week, since last call or visit	Num
97	g17days	number of hypoglycemic episodes (SMBG ¡70 mg/dL or ¡3.9 mmol/L) in last 7 days	Num
98	g1_report	hypoglycemia requiring hospitalization, emergency care without hospital admission, or medical assistance without emergency care or hospitalization, number of times since last call or visit	Num
99	g2hwofch	frequency of use of CHO/Insulin ratio	Num
100	g2hwofba	frequency of insulin injection/inhalation as prescribed for basal (background) insulin	Num
101	g2hwofbo	frequency of insulin injection/inhalation as prescribed for injected bolus (premeal) insulin	Num
102	g2aveba	visit entry total basal insulin/day	Num
103	g2avebol	visit entry total bolus insulin/day	Num
104	g2avetid	visit entry total injected insulins/day	Num
		Data Set Name: f08_09_glycemiamanagement.sas7bdat	
105	HoursAll	average hrs/week spent in any activity	Num

Continued on next page

Table 3 – continued from previous page

	Variables	Description	Type
106	HoursMod	average hrs/week spent in moderate activity	Num
		Data Set Name: f29_champsphysicalactivity.sas7bdat	
107	x3malb	micro or macro albuminuria within past 2 years	Num
		Data Set Name: f01_inclusionexclusionsummary.sas7bdat	
108	total_mmse	MMSE Total Score	Num
109	total_dsc	DSST Total Score	Num
110	stroop	STROOP Interference Score	Num
111	ravlt	RAVLT Score	Num
		Data Set Name: mind.sas7bdat	
112	ses03	participant experienced being thirsty during the past month	Num
113	Ses51	participant experienced pains in legs or calves when walking during the past month	Num
		Data Set Name: f23_hrql.sas7bdat	
114	ateskfat	how often participant ate raw vegetables or fresh fruit when snacking	Num
115	Aterdmt	participant ate red meat such as beef, pork, or lamb in last 3 months	Num
116	Substitution	DIET Factor 1: Average score for low-fat choices	Num
		Data Set Name: f26_dietquestionnaire.sas7bdat	

Table 4: Characteristics of selected features according to follow-up in ACCORD dataset. mean ± standard deviation for continuous and count (percentage %) for the categorical variables.

Demographics	Values
Age	62.70 ± 6.59
BMI (kg/m^2)	32.22 ± 5.39
Female	496 (42.8%)
Fasting Glucose	175.28 ± 56.11
HbA1c	8.30 ± 1.05
g1check	18.57 ± 6.46
g1diabed	18.46 ± 12.30
g1nutrit	13.09 ± 9.63
Sulfonylurea	520 (44.22%)
Meglitinide	31 (2.64%)
Reg Insulin	239 (20.32%)
La Insulin	178 (15.14%)
Othbol Insulin	44 (3.74%)
Premix Insulin	212 (18.03%)
NPH or L Insulin	594 (50.51%)

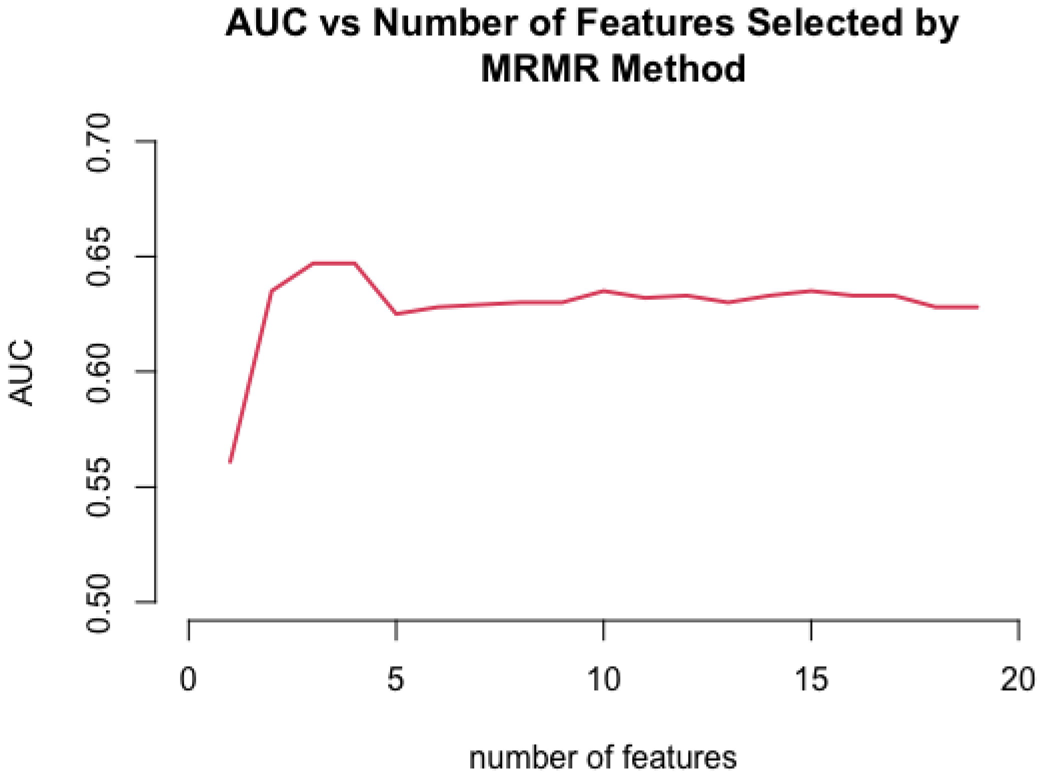

Figure 2: Area Under Curve (AUC) versus selected features by MRMR method. The number of features should be specified in the MRMR code for entire ACCORD data. Therefore, we need to define how many features should be selected by MRMR method in advance. To do this, MRMR selected features from 1 to 17, then we calculated the AUC value for each. Finally, we obtained the highest AUC for the number of 4 features.

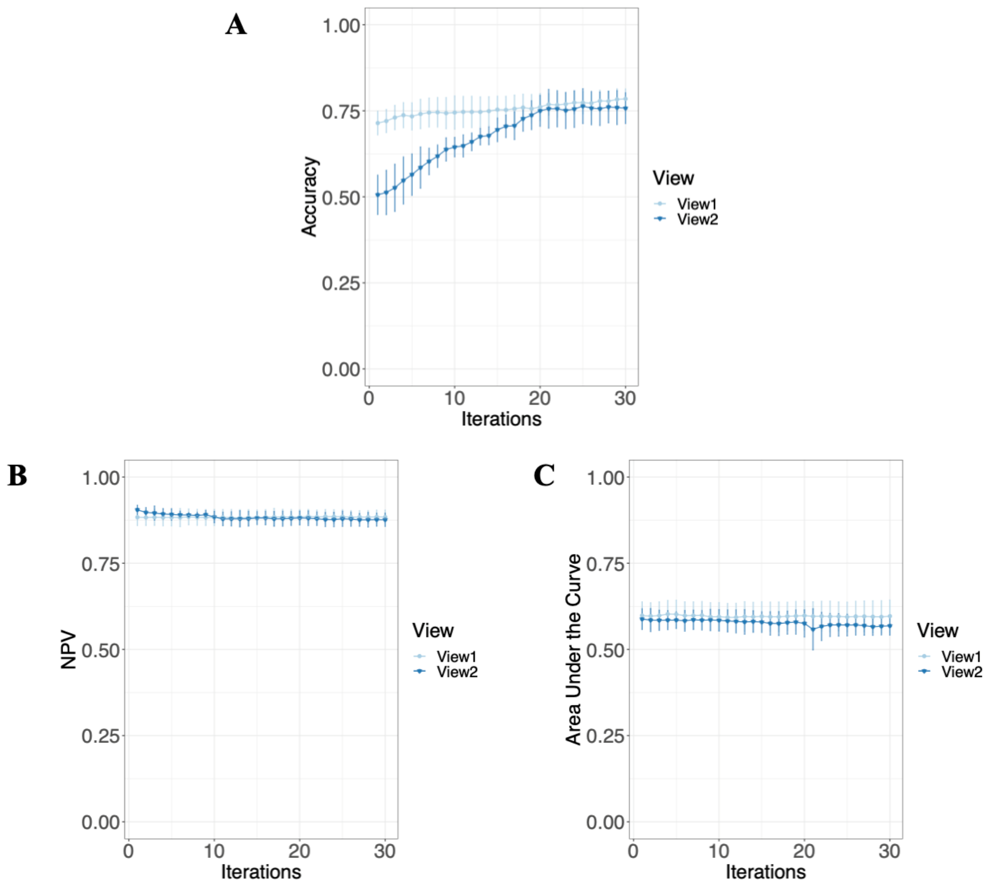

Figure 3: Mean accuracy metrics of test results for each iteration in multi-view co-training for NB MD features. We split the data into two different labeled data as View 1 and View 2. From the unlabeled data, we created two pools of datasets: U1 and U2. Following the training process, predictions are generated for both U1 and U2, and pseudo labels are created. The iteration repeated thirty times. (5 negative and 1 positive) (A) Accuracy, (B) NPV, (C) AUC-ROC

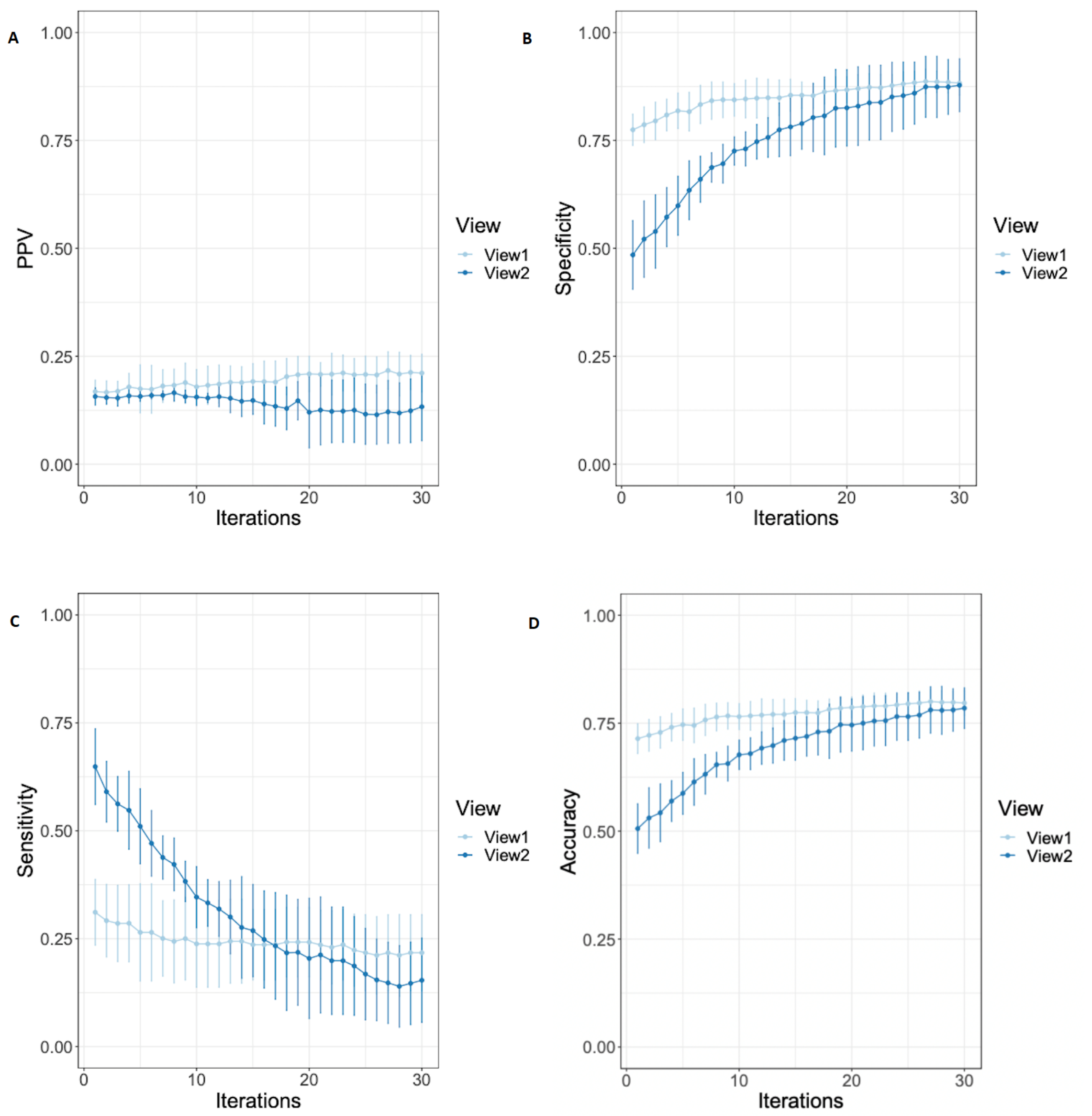

Figure 4: Mean accuracy metrics of test results for each iteration in multi-view co-training for NB MD features. We split the data into two different labeled data as View 1 and View 2. From the unlabeled data, we created two pools of datasets: U1 and U2. Following the training process, predictions are generated for both U1 and U2, and pseudo labels are created. The iteration repeated thirty times. (7 negative and 1 positive) (a) PPV, (b) Specificity, (c) Sensitivity, (d) Accuracy.

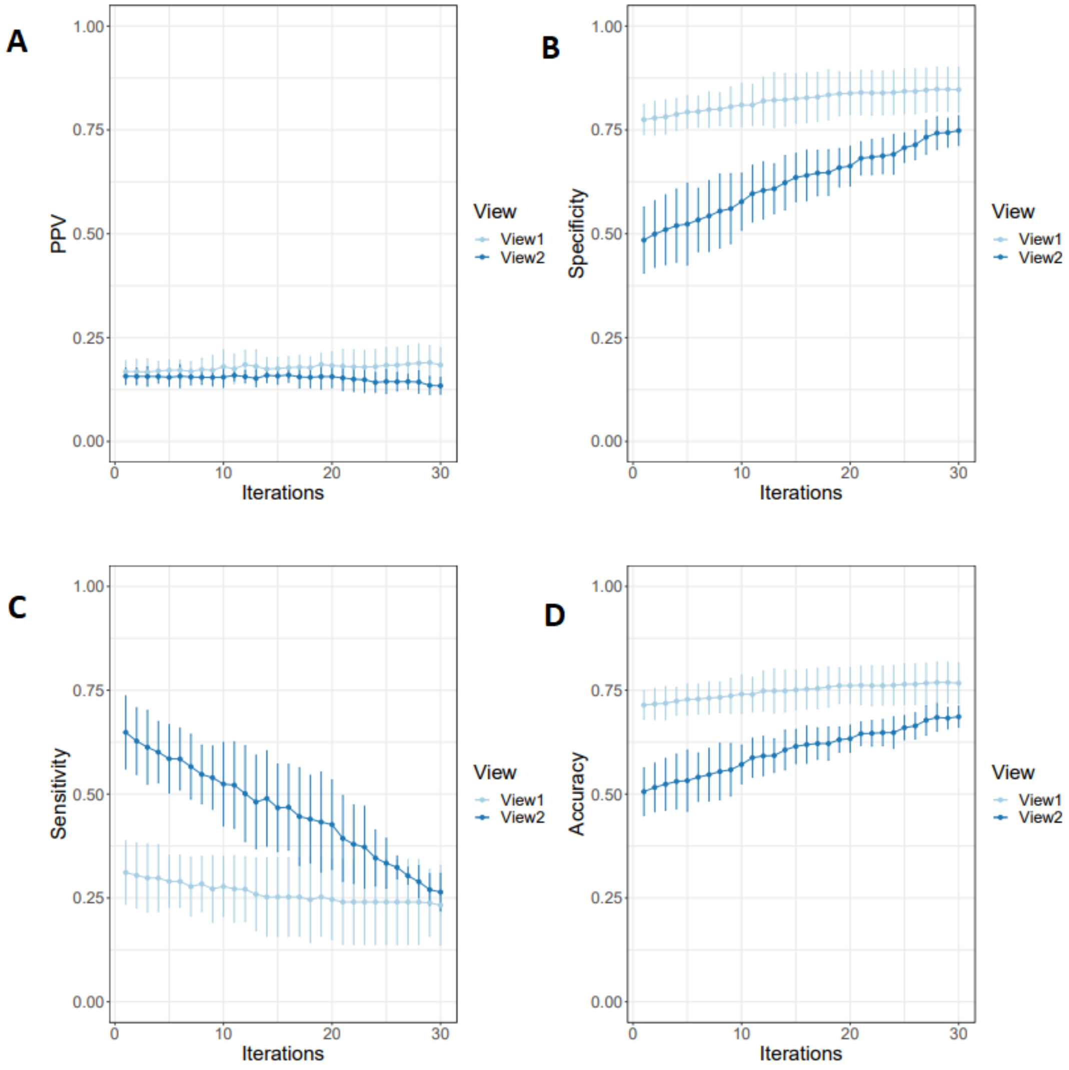

Figure 5: Mean accuracy metrics of test results for each iteration in multi-view co-training for NB MD features. We split the data into two different labeled data as View 1 and View 2. From the unlabeled data, we created two pools of datasets: U1 and U2. Following the training process, predictions are generated for both U1 and U2, and pseudo labels are created. The iteration repeated thirty times. (3 negative and 1 positive) (a) PPV, (b) Specificity, (c) Sensitivity, (d) Accuracy.

Table 5: Comparing multi-view co-training model results on MD and MRMR features with under-sampling imbalanced solution MD Variables and MRMR (7 negative and 1 positive).

	NB											
	MD						MRMR					
Iterations	NPV	PPV	Spec	Sens	Acc	F	NPV	PPV	Spec	Sens	Acc	F
View1 1st iteration	0.883	0.168	0.775	0.311	0.714	0.217	0.887	0.192	0.804	0.314	0.740	0.233
View1 last iteration	0.884	0.211	0.883	0.217	0.797	0.211	0.883	0.202	0.880	0.212	0.793	0.204
Percentage	(0.07%)	(25.45%)	(13.96%)	(-30.11%)	(-11.54%)	(2.71%)	(-0.52%)	(5.23%)	(9.43%)	(-32.36%)	(7.24%)	(-12.64%)
View2 1st iteration	0.905	0.157	0.485	0.649	0.506	0.252	0.910	0.172	0.570	0.609	0.575	0.267
View3 last iteration	0.876	0.133	0.878	0.154	0.785	0.177	0.876	0.146	0.887	0.141	0.793	0.174
Percentage	(-3.23%)	(-15.17%)	(81.07%)	(-76.31%)	(55.15%)	(-29.81%)	(-3.74%)	(-15.17%)	(55.65%)	(-76.89%)	(-37.88%)	(-34.98%)
	MD						MRMR					
Iterations	NPV	PPV	Spec	Sens	Acc	F	NPV	PPV	Spec	Sens	Acc	F
View1 1st iteration	0.898	0.162	0.565	0.568	0.565	0.251	0.899	0.159	0.544	0.586	0.549	0.249
View1 last iteration	0.887	0.209	0.843	0.276	0.770	0.237	0.886	0.187	0.812	0.295	0.745	0.227
Percentage	(-1.27%)	(28.60%)	(49.04%)	(-51.39%)	(36.29%)	(-5.53%)	(-1.37%)	(17.65%)	(49.15%)	(-49.61%)	(35.59%)	(-8.94%)
View2 1st iteration	0.905	0.162	0.528	0.618	0.540	0.256	0.931	0.161	0.393	0.785	0.444	0.266
View2 last iteration	0.879	0.183	0.879	0.183	0.789	0.180	0.872	NA	1.000	0.000	0.872	NA
Percentage	(-2.85%)	(13.18%)	(66.64%)	(-70.46%)	(46.14%)	(-29.84%)	(-6.42%)	NA	(154.60%)	(-100.00%)	(96.36%)	NA

Table 6: Results of combining views using both AND, and OR rules selected for 7 Negative and 1 positive pseudo labels.

	AND Rule					
	NPV	PPV	Spec	Sens	Acc	F
NB MD	0.872	0.143	0.988	0.013	0.863	0.024
NB MRMR	0.871	0.091	0.981	0.013	0.856	0.023
RF MD	0.874	0.240	0.982	0.040	0.861	0.068
RF MRMR	0.872	NA	1.000	0.000	0.872	NA
	OR Rule					
	NPV	PPV	Spec	Sens	Acc	F
NB MD	0.890	0.185	0.773	0.351	0.719	0.243
NB MRMR	0.890	0.190	0.787	0.338	0.730	0.243
RF MD	0.896	0.193	0.742	0.417	0.701	0.264
RF MRMR	0.886	0.186	0.812	0.291	0.745	0.227

Table 7: Comparing multi-view co-training model results on MD and MRMR selected data with undersampling imbalanced solution. Results of the MD data and MRMR data (3 negative and 1 positive).

	NB											
	MD						MRMR					
Iterations	NPV	PPV	Spec	Sens	Acc	F	NPV	PPV	Spec	Sens	Acc	F
View1 1st iteration	0.883	0.168	0.775	0.311	0.714	0.217	0.887	0.192	0.804	0.314	0.740	0.233
View1 last iteration	0.881	0.184	0.847	0.232	0.767	0.200	0.883	0.192	0.845	0.247	0.767	0.210
Percentage	(-0.24%)	(9.35%)	(9.302%)	(-25.30%)	(7.38%)	(-7.41%)	(-0.516%)	(-0.06%)	(5.06%)	(-21.35%)	(3.68%)	(-9.92%)
View2 1st iteration	0.905	0.157	0.485	0.649	0.506	0.252	0.910	0.172	0.570	0.609	0.575	0.267
View2 last iteration	0.874	0.134	0.748	0.264	0.686	0.177	0.883	0.141	0.722	0.340	0.673	0.198
Percentage	(-3.45%)	(-14.62%)	(54.36%)	(-59.31%)	(35.65%)	(-29.80%)	(-2.96%)	(-18.06%)	(26.60%)	(-44.14%)	(17.16%)	(-26.02%)
	MD						MRMR					
Iterations	NPV	PPV	Spec	Sens	Acc	F	NPV	PPV	Spec	Sens	Acc	F
1st iteration	0.898	0.162	0.564	0.568	0.564	0.251	0.897	0.157	0.543	0.579	0.548	0.246
Last	0.894	0.177	0.698	0.444	0.664	0.252	0.883	0.148	0.659	0.407	0.626	0.216
Percentage	(-0.46%)	(9.04%)	(23.60%)	(-21.83%)	(17.79%)	(0.45%)	(-1.61%)	(-5.53%)	(21.19%)	(-29.70%)	(14.29%)	(-12.13%)
View1 1st iteration	0.905	0.162	0.529	0.618	0.541	0.256	0.931	0.161	0.392	0.785	0.443	0.266
View1 last iteration	0.887	0.162	0.678	0.418	0.645	0.233	0.879	0.123	0.785	0.266	0.717	0.209
Percentage	(-1.95%)	(0.185%)	(28.32%)	(-32.35%)	(19.19%)	(-9.243%)	(-5.62%)	(-23.75%)	(100.27%)	(-66.10%)	(61.79%)	(-21.55%)

Table 8: Results of combining views using both AND, and OR rules selected for 3 Negative and 1 positive pseudo labels.

	AND Rule					
	NPV	PPV	Spec	Sens	Acc	F
NB MD	0.872	0.128	0.960	0.040	0.842	0.061
NB MRMR	0.876	0.194	0.943	0.093	0.834	0.126
RF MD	0.882	0.209	0.900	0.179	0.808	0.193
RF MRMR	0.880	0.214	0.921	0.146	0.821	0.173
	OR Rule					
	NPV	PPV	Spec	Sens	Acc	F
NB MD	0.887	0.154	0.635	0.450	0.611	0.229
NB MRMR	0.892	0.161	0.623	0.490	0.606	0.242
RF MD	0.910	0.161	0.475	0.682	0.502	0.260
RF MRMR	0.880	0.137	0.522	0.517	0.521	0.217

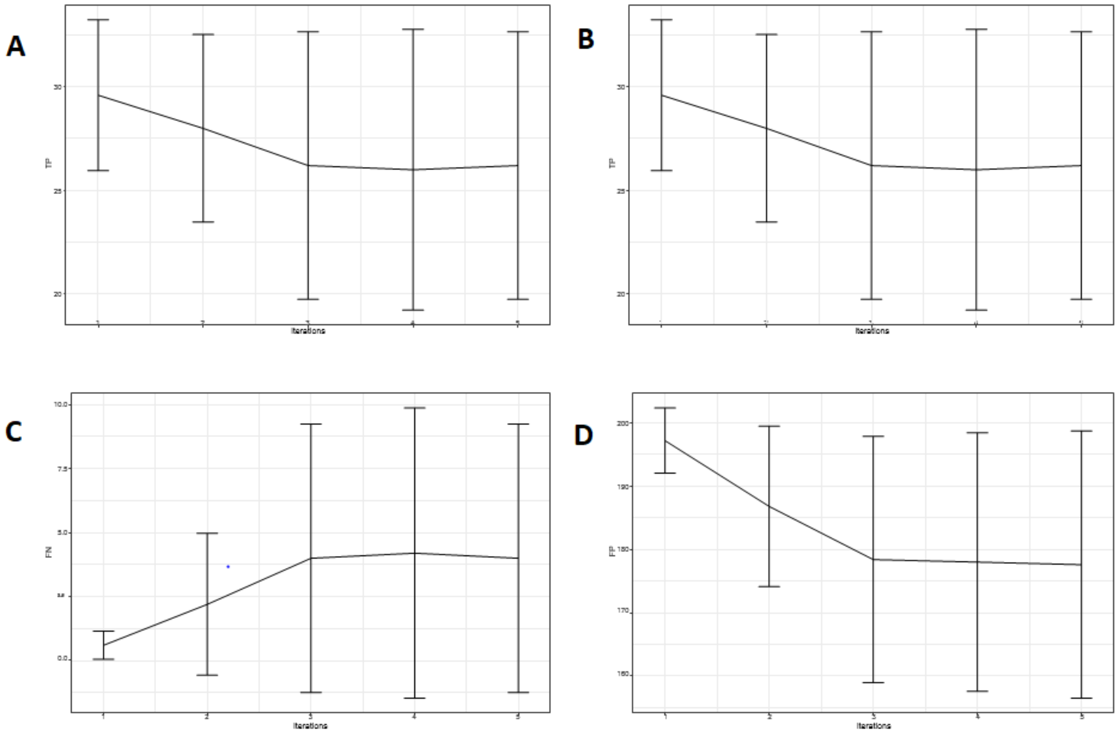

Figure 6: Confusion matrix for single-view co-training (A) TP, (B) TN, (C) FN, (D)FP.

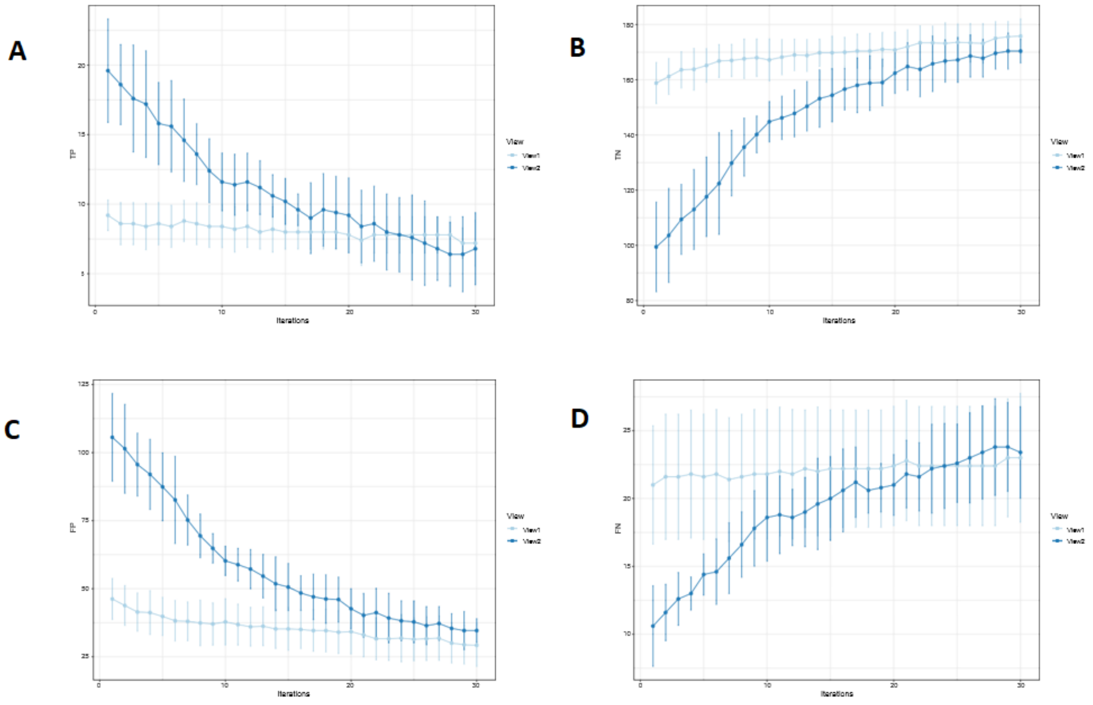

Figure 7: Confusion matrix for multi-view co-training (A) TP, (B) TN, (C) FN, (D) FP.

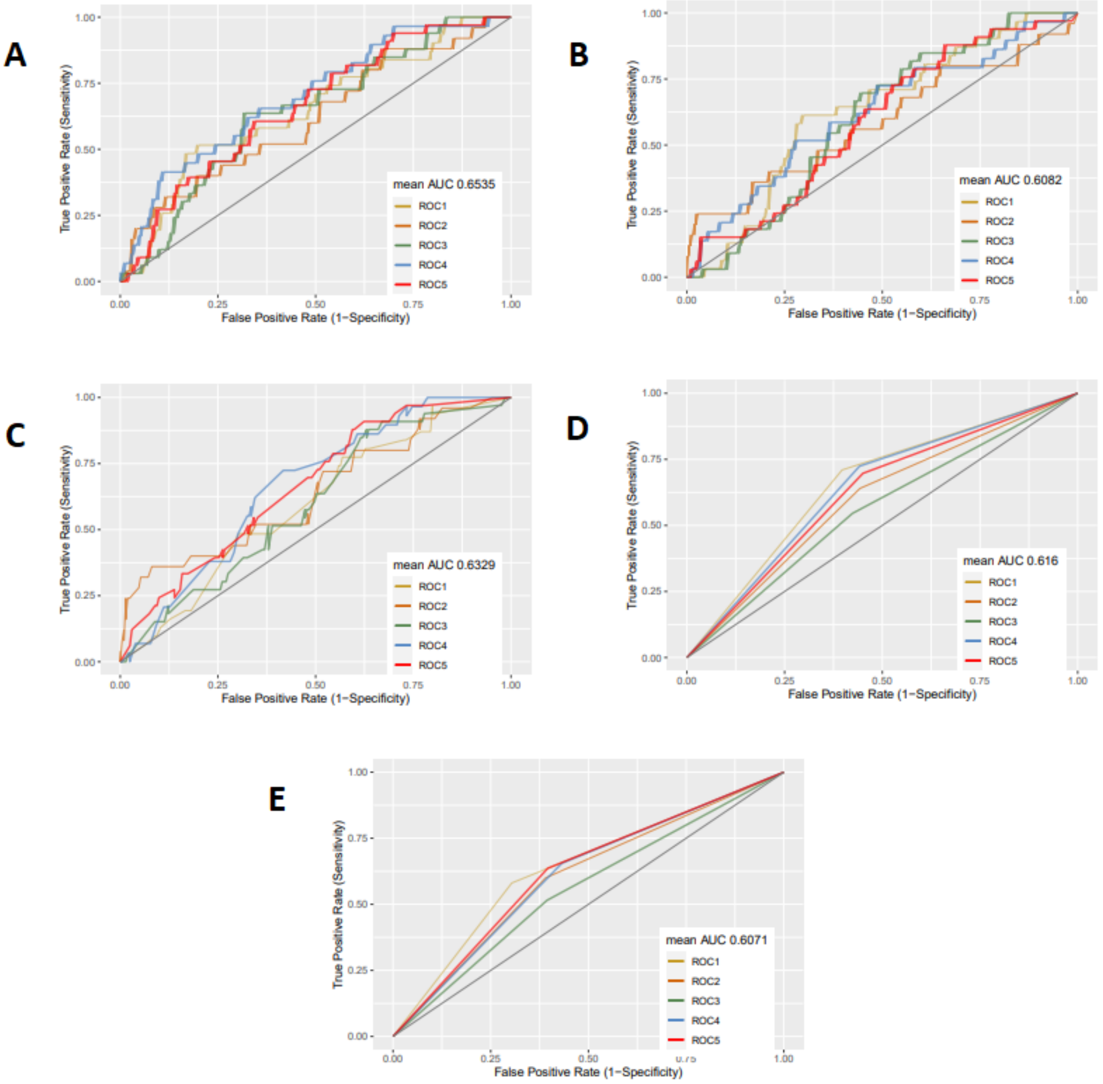

Figure 8: 5-fold ROC curves of conventional machine learning algorithms for (A) LR, (B) NB, (C) XGBoost, (D) RF, (E) SVM.

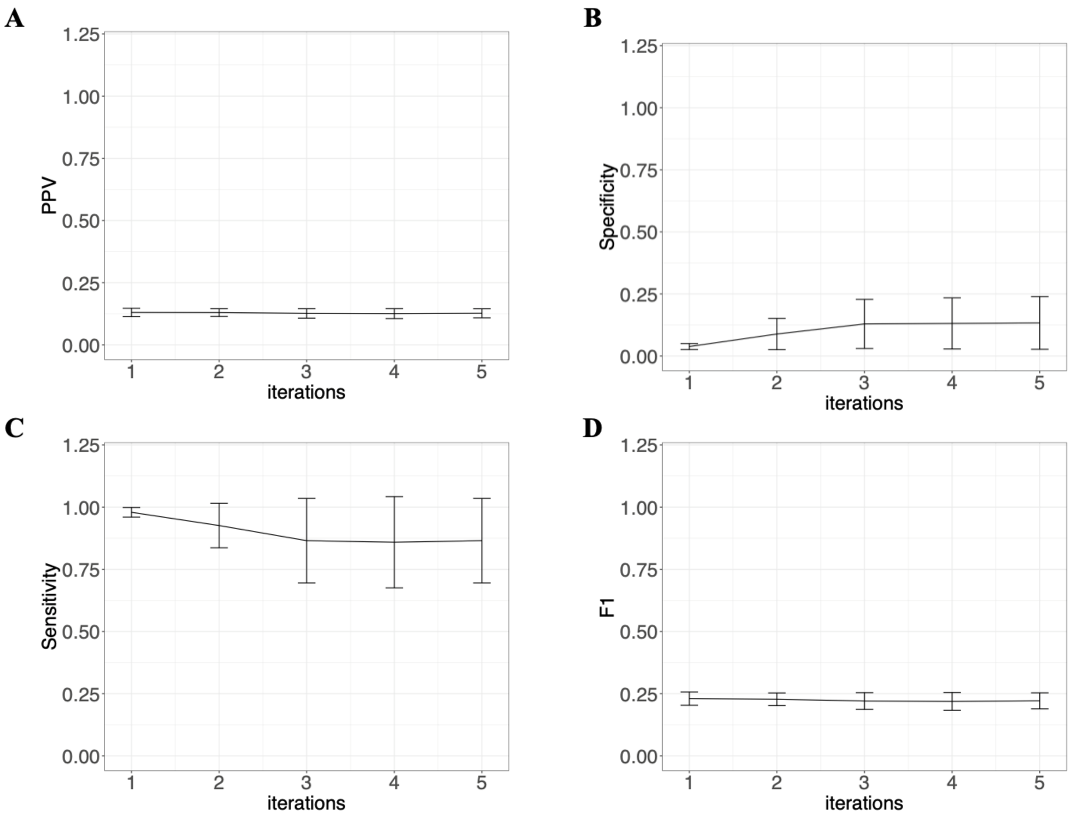

Figure 9: Mean accuracy metrics of test results for each iteration in single-view co-training for NB using MD features. The error bars show the mean ± standard deviations. We first split the labeled data as train and test set, then train the labeled data with the naive Bayes model and after that, we create the pseudo labels by estimating the unlabeled data and selecting the most confident labels, corresponding to probabilities higher than 0.9. This procedure is repeated until there is no unlabeled data to be selected. The figure shows the performance metrics of the test data results as (A) Positive Predicted Value (PPV), (B) Specificity, (C) Sensitivity, (D) F1-score (F1).

Table 9: Main features: The 17 features used in our study.

Variables	Description
hba1c mean	Mean of HbA1c values
hba1c std	Standard deviation of HbA1c values
fpg mean	Mean of fasting plasma glucose values
fpg std	Standard deviation of fasting plasma glucose values
g1check mean	Mean of average frequency of blood sugar check
g1check std	Standard deviation of average frequency of blood sugar check
g1diabed mean	Mean of general diabetes education
g1diabed std	Standard deviation of general diabetes education
g1nutrit mean	Mean of nutrition education
g1nutrit std	Standard deviation of nutrition education
sulfonylurea mean	Mean of sulfonylureas
meglitinide mean	Mean of meglitinides
nphl mean	Mean of NPH or L insulins
reg insulin mean	Mean of regular insulins
la insulin mean	Mean of lispro or aspart insulins
othbol insulin mean	Mean of other bolus insulins
premix insulin mean	Mean of premixed insulins

Feature Selection Methods

We first created all 118 candidate risk factors of SH events, which are listed in the supplemental Table 9. Afterwards, a down selection of the most significant variables was made by medical doctors from the Endocrinology Department at Beth Israel Medical Center. The doctors selected the top-12 features out of the 118 candidate risk factors. As the ACCORD study is a follow-up study, we computed the mean and standard deviations of the observations. This process resulted in 17 variables for further analysis, as represented in Table 9.

Least Absolute Shrinkage and selection operator (LASSO)

LASSO is a regression technique that uses the following loss function for both regularization and feature selection,

$$\text{Loss} = \sum_{i=1}^{N} \left(\left| y_i - \sum_j \beta_j x_{ij} \right|^2 + \lambda \sum_j \beta_j \right) \tag{1}$$

where $\lambda \sum_j \beta_j < t$ is the L_1 norm of the β parameter vector and λ is a non-negative parameter $\sum_j \beta_j$ controlling the degree of regularization. The ACCORD dataset consists of 118 variables. We created a potential estimation variable list by selecting 12 variables from the ACCORD dataset. Subsequently, we applied the LASSO feature selection to the dataset and compared the results obtained from these variables with those from the MD data and the other feature selection algorithm results. Finally, the features selected according to this method are FPG std, HbA1c mean, g1diabed std, NPHL Insulin, FPG mean, g1nutrit std, Othbol Insulin, Sulfonylurea mean, and Premix Insulin mean.

Boruta

Boruta is a feature selection method that uses a wrapper method built in the RF classifier. The Boruta method selects the features by following the steps below. 1.Synthetic new variables, called shadow

features, are generated by randomly shuffling each variable. Shadow features are concatenated with the original dataset. 2.RF algorithm is run on X on y and Z scores are computed. 3.Calculate the maximum Z-score (MZSA) within the shadow features. 4.Original variables that are significantly lower than this MZSA are marked as unimportant. Original variables that are significantly higher than MZSA are marked as important. 5.Shadow features are deleted. 6. The algorithm is repeated until all variables are marked as important or unimportant, or until the constraint of the RF algorithm persists. We applied the Boruta feature selection method to the data and compared the results obtained from these variables with those from the MD data. Finally, the features selected according to this method are FPG std, HbA1c mean, g1diabed std, NPHL Insulin, FPG mean, g1nutrit std, Othbol Insulin, and g1 nutrition mean.

Maximum Relevance-Minimum Redundancy (MRMR).

This method maximizes the relevance of selected features while minimizing their redundancy. Since FCQ method in MRMR is the simplest, fastest and more effective than other methods, we focus on this method in this article. The MRMR method first calculates the maximum relevance between X and y and minimum redundancy between covariates itself, then by dividing the relevance to redundancy it calculates the FCQ score. Relevance is computed by the $F(X, y)$-statistics between X and y. Redundancy is calculated by correlation between the X_i. So FCQ score (f^{FCQ}) can be calculated by the following formula:

$$f^{FCQ} = \frac{F(X_i, y)}{\frac{1}{|S|}\sum_{X_s \in S} \rho(X_s, X_i)} \tag{2}$$

1. Specify the number of features K we want to select.
2. Compute the F-statistics between X_i and y.
3. Rank the F-statistics and select the best feature which has the highest F-statistics.
4. While (number of selected features $< K$):
   (a) Calculate correlation between selected features and unselected features.
   (b) Calculate f^{FCQ} by using Equation 2.
   (c) Add the variable, which has the highest f^{FCQ} score.

We applied the Maximum Relevance Minimum Redundancy (MRMR) feature selection method to the data and compared the results obtained from these variables with those from the MD data. Before applying the MRMR method, it is necessary to specify how many features will be selected. Therefore, K = 1 to 17 were first defined and the MRMR method for each K was applied to the data. AUC values from each feature were recorded and plotted as shown in SM Fig. 2. Finally, the features selected according to this method were FPG STD, HbA1c Mean, g1diabed STD, and NPHL Insulin Mean variables.

Performance Measures

The ACCORD dataset consists of various independent variables like continuous and categorical, and the response variables consist of binary categorical variables. Hence, Sensitivity, (or Recall), Specificity, Positive Predictive Values (PPV), (or Precision), Negative Predictive Values (NPV), Accuracy and F1 Score (harmonic mean of sensitivity and precision) are selected below to evaluate the performance of the models.

$$\text{Specificity} = \frac{TN}{TN + FP} \tag{3}$$

$$\text{PPV} = \frac{TP}{TP + FP} \tag{4}$$

$$\text{NPV} = \frac{TN}{TN + FN} \tag{5}$$

$$\text{Sensitivity} = \frac{TP}{TP + FN} \tag{6}$$

$$\text{Accuracy} = \frac{TP + TN}{TP + TN + FP + FN} \tag{7}$$

$$\text{F1-score} = \frac{2 \times \text{PPV} \times \text{Sensitivity}}{\text{PPV} + \text{Sensitivity}} \tag{8}$$

Equations 3–8 denote the number of correctly categorized positive labels by TP (the true positive) and the number of correctly classified negative labels by TN (the genuine negative). The number of labels expected to be negative but actually positive predicted is indicated by FP (false positive), while FN (false negative) indicates labels that are predicted negatively but are actually expected to be positive.

Algorithmic Representation of the Multi-View Co-Training Model

1. Initialize

- **Input**: Dataset with labeled and unlabeled data.
- **Output**: Predictions for severe hypoglycemia events.
- **Parameters**:
  - p: Number of positive pseudo-labels to add per iteration.
  - n: Number of negative pseudo-labels to add per iteration.
  - k: Number of iterations.

2. Preprocess Data

- Perform data cleaning and handling of missing values.
- Normalize or standardize features if necessary.

3. Feature Selection

- Apply multiple feature selection techniques:
  - Medical preselection by domain experts.
  - LASSO (Least Absolute Shrinkage and Selection Operator).
  - Boruta: A random forest-based feature selection method.
  - MRMR (Minimum Redundancy Maximum Relevance).
- Consolidate features selected by all methods for robustness.

4. **Split the Dataset**
   - Divide the data into training and test subsets.
   - Ensure both subsets are representative of the overall dataset.

5. **Initialize Models for Each View**
   - **Model 1 (View 1)**: Focus on basic clinical data.
   - **Model 2 (View 2)**: Focus on detailed metabolic parameters.
   - Each model is initialized separately but shares underlying assumptions.

6. **Co-Training Loop**

For each iteration from 1 to k:

- **6.1 Train each model on its respective view** using available labeled data.
- **6.2 Apply models to the unlabeled dataset** to predict pseudo-labels.
- **6.3 Select the most confident pseudo-labels**:
  - p positive and n negative labels per model based on the highest prediction confidence.
- **6.4 Add these labels to the training set** for the next iteration.
- **6.5 Evaluate model performance** on a separate validation set or through cross-validation within the training set.

7. **Combine Predictions from Both Views**
   - Use ensemble techniques like averaging or voting to combine predictions:
     - **AND Rule**: Predict positive only if both models agree on positive.
     - **OR Rule**: Predict positive if either model predicts positive.

8. **Final Evaluation**
   - Assess the final model on the test set with metrics such as accuracy, sensitivity, specificity, and F1-score.
   - Interpret the results and adjust parameters if necessary.

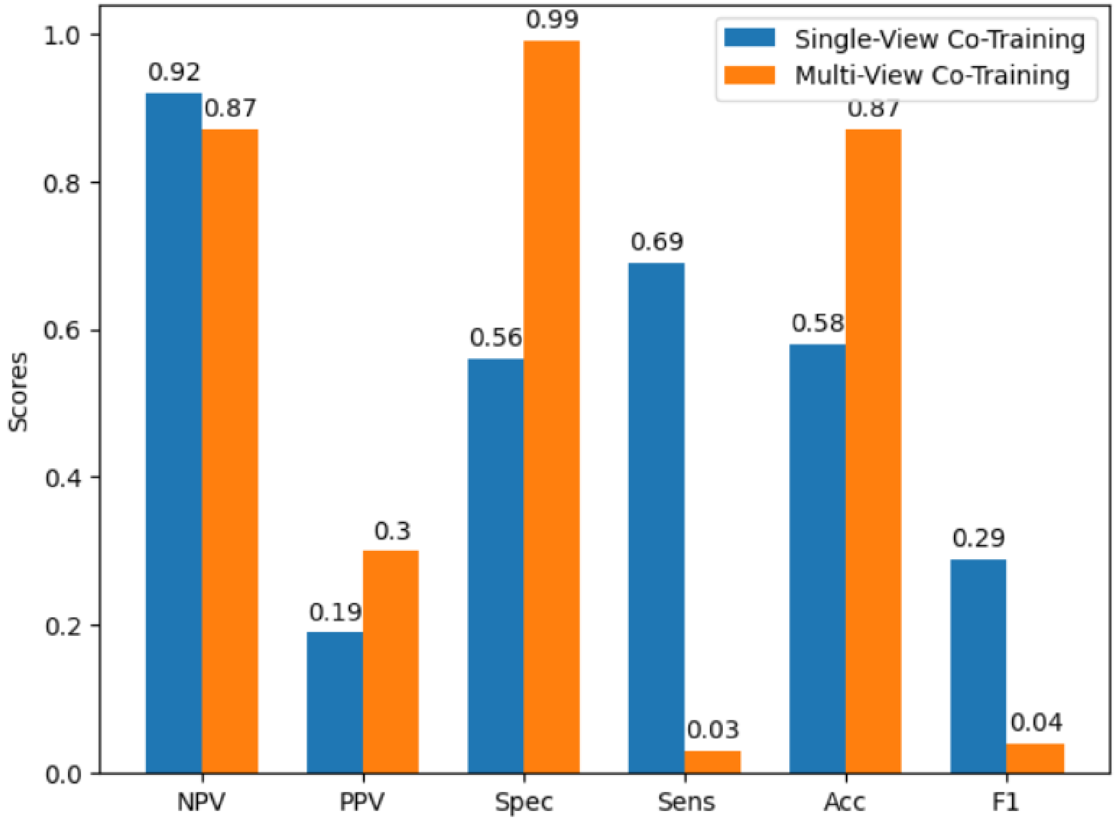

Figure 10: Performance comparison between Single-View and Multi-View Co-Training Models.
